# Multiple Loci Are Associated with White Blood Cell Phenotypes

**DOI:** 10.1371/journal.pgen.1002113

**Published:** 2011-06-30

**Authors:** Michael A. Nalls, David J. Couper, Toshiko Tanaka, Frank J. A. van Rooij, Ming-Huei Chen, Albert V. Smith, Daniela Toniolo, Neil A. Zakai, Qiong Yang, Andreas Greinacher, Andrew R. Wood, Melissa Garcia, Paolo Gasparini, Yongmei Liu, Thomas Lumley, Aaron R. Folsom, Alex P. Reiner, Christian Gieger, Vasiliki Lagou, Janine F. Felix, Henry Völzke, Natalia A. Gouskova, Alessandro Biffi, Angela Döring, Uwe Völker, Sean Chong, Kerri L. Wiggins, Augusto Rendon, Abbas Dehghan, Matt Moore, Kent Taylor, James G. Wilson, Guillaume Lettre, Albert Hofman, Joshua C. Bis, Nicola Pirastu, Caroline S. Fox, Christa Meisinger, Jennifer Sambrook, Sampath Arepalli, Matthias Nauck, Holger Prokisch, Jonathan Stephens, Nicole L. Glazer, L. Adrienne Cupples, Yukinori Okada, Atsushi Takahashi, Yoichiro Kamatani, Koichi Matsuda, Tatsuhiko Tsunoda, Toshihiro Tanaka, Michiaki Kubo, Yusuke Nakamura, Kazuhiko Yamamoto, Naoyuki Kamatani, Michael Stumvoll, Anke Tönjes, Inga Prokopenko, Thomas Illig, Kushang V. Patel, Stephen F. Garner, Brigitte Kuhnel, Massimo Mangino, Ben A. Oostra, Swee Lay Thein, Josef Coresh, H.-Erich Wichmann, Stephan Menzel, JingPing Lin, Giorgio Pistis, André G. Uitterlinden, Tim D. Spector, Alexander Teumer, Gudny Eiriksdottir, Vilmundur Gudnason, Stefania Bandinelli, Timothy M. Frayling, Aravinda Chakravarti, Cornelia M. van Duijn, David Melzer, Willem H. Ouwehand, Daniel Levy, Eric Boerwinkle, Andrew B. Singleton, Dena G. Hernandez, Dan L. Longo, Nicole Soranzo, Jacqueline C. M. Witteman, Bruce M. Psaty, Luigi Ferrucci, Tamara B. Harris, Christopher J. O'Donnell, Santhi K. Ganesh

**Affiliations:** 1 Laboratory of Neurogenetics, Intramural Research Program, National Institute on Aging (NIA), National Institutes of Health (NIH), Bethesda, Maryland, United States of America; 2 Collaborative Studies Coordinating Center, Department of Biostatistics, University of North Carolina at Chapel Hill, Chapel Hill, North Carolina, United States of America; 3 Longitudinal Studies Section, Clinical Research Branch, NIA, NIH, Baltimore, Maryland, United States of America; 4 Department of Epidemiology, Erasmus MC, Rotterdam, The Netherlands; 5 Netherlands Consortium for Healthy Aging (NGI-NCHA), The Netherlands Genomics Initiative, Leiden, The Netherlands; 6 National Heart, Lung, and Blood Institute's Framingham Heart Study, Framingham, Massachusetts, United States of America; 7 Department of Neurology, Boston University School of Medicine, Boston, Massachusetts, United States of America; 8 Icelandic Heart Association, Kopavogur, Iceland; 9 University of Iceland, Reykjavik, Iceland; 10 Division of Genetics and Cell Biology, San Raffaele Scientific Institute, Milano, Italy; 11 Institute of Molecular Genetics–CNR, Pavia, Italy; 12 Department of Medicine University of Vermont College of Medicine, Burlington, Vermont, United States of America; 13 Department of Pathology University of Vermont College of Medicine, Burlington, Vermont, United States of America; 14 Department of Biostatistics, Boston University School of Public Health, Boston, Massachusetts, United States of America; 15 Institute of Immunology and Transfusion Medicine, Ernst-Moritz-Arndt-University Greifswald, Greifswald, Germany; 16 Genetics of Complex Traits, Peninsula College of Medicine and Dentistry, University of Exeter, United Kingdom; 17 Laboratory for Epidemiology, Demography, and Biometry, NIA, NIH, Bethesda, Maryland, United States of America; 18 Medical Genetics, IRCCS–Burlo Garofolo/University of Trieste, Trieste, Italy; 19 Department of Epidemiology and Prevention, Division of Public Health Sciences, Wake Forest University, Winston-Salem, North Carolina, United States of America; 20 Department of Biostatistics, University of Washington, Seattle, Washington, United States of America; 21 Division of Epidemiology and Community Health, University of Minnesota, Minneapolis, Minnesota, United States of America; 22 Department of Epidemiology, University of Washington, Seattle, Washington, United States of America; 23 Institute of Genetic Epidemiology, Helmholtz Zentrum München, German Research Center for Environmental Health, Neuherberg, Germany; 24 Wellcome Trust Centre for Human Genetics, University of Oxford, Oxford, United Kingdom; 25 Oxford Centre for Diabetes, Endocrinology and Metabolism, University of Oxford, Oxford, United Kingdom; 26 Division of Clinical Epidemiology and Aging Research, German Cancer Research Center, Heidelberg, Germany; 27 Community Medicine, Ernst-Moritz-Arndt-University Greifswald, Greifswald, Germany; 28 University of North Carolina, School of Public Health, United States of America; 29 Center for Human Genetic Research, Department of Neurology, Massachusetts General Hospital, Boston, Massachusetts, United States of America; 30 Program in Medical and Population Genetics, Broad Institute, Cambridge, Massachusetts, United States of America; 31 Institute of Epidemiology I, Helmholtz Zentrum München, German Research Center for Environmental Health, Neuherberg, Germany; 32 Institute of Epidemiology II, Helmholtz Zentrum München, German Research Center for Environmental Health, Neuherberg, Germany; 33 Interfaculty Institute for Genetics and Functional Genomics, Ernst-Moritz-Arndt-University Greifswald, Greifswald, Germany; 34 Cardiovascular Health Resarch Unit and Department of Medicine, University of Washington, Seattle, Washington, United States of America; 35 Department of Haematology, University of Cambridge and National Health Service Blood and Transplant, Cambridge, United Kingdom; 36 Medical Genetics Institute, Cedars-Sinai Medical Center, Los Angeles, California, United States of America; 37 Department of Physiology and Biophysics, University of Mississippi Medical Center, Jackson, Mississippi, United States of America; 38 Montreal Heart Institute and Universite de Montreal, Montreal, Canada; 39 Division of Endocrinology, Brigham and Women's Hospital and Harvard Medical School, Boston, United States of America; 40 Institute for Clinical Chemistry and Laboratory Medicine, Ernst-Moritz-Arndt-University Greifswald, Greifswald, Germany; 41 Institute of Human Genetics, Klinikum rechts der Isar, Technical University Munich, Munich, Germany; 42 Institute of Human Genetics, Helmholtz Zentrum München, German Research Center for Environmental Health, Neuherberg, Germany; 43 Laboratory for Statistical Analysis, Center for Genomic Medicine (CGM), Institute of Physical and Chemical Research (RIKEN), Yokohama, Japan; 44 Department of Allergy and Rheumatology, Graduate School of Medicine, University of Tokyo, Tokyo, Japan; 45 Centre d'Etude du Polymorphisme Humain (CEPH), Paris, France; 46 Laboratory of Molecular Medicine, Human Genome Center, Institute of Medical Science, University of Tokyo, Tokyo, Japan; 47 Laboratory for Medical Informatics, CGM, RIKEN, Yokohama, Japan; 48 Laboratory for Cardiovascular Diseases, CGM, RIKEN, Yokohama, Japan; 49 Laboratory for Genotyping Development, CGM, RIKEN, Yokohama, Japan; 50 Department of Medicine, University of Leipzig, Leipzig, Germany; 51 LIFE Study Centre, University of Leipzig, Leipzig, Germany; 52 Unit for Molecular Epidemiology, Helmholtz Zentrum München, German Research Center for Environmental Health, Neuherberg, Germany; 53 Department of Twin Research and Genetic Epidemiology, King's College London, London, United Kingdom; 54 Department of Clinical Genetics, Erasmus MC, Rotterdam, The Netherlands; 55 Molecular Haematology, King's College London, London, United Kingdom; 56 Department of Epidemiology, Johns Hopkins University, Baltimore, Maryland, United States of America; 57 Institute of Medical Informatics, Biometry and Epidemiology, Chair of Epidemiology, Ludwig-Maximilians-Universität, Munich, Germany; 58 Klinikum Grosshadern, Munich, Germany; 59 Office of Biostatistical Research, Division of Cardiovascular Sciences, NHLBI, NIH, Bethesda, Maryland, United States of America; 60 Department of Internal Medicine, Erasmus MC, Rotterdam, The Netherlands; 61 Geriatric Unit ASF, Firenze, Italy; 62 McKusick-Nathans Institute of Genetic Medicine, Johns Hopkins University, Baltimore, Maryland, United States of America; 63 Epidemiology and Public Health, Peninsula College of Medicine and Dentistry, University of Exeter, Exeter, United Kingdom; 64 The European Centre for Environment and Human Health, PCMD, Truro, United Kingdom; 65 Wellcome Trust Sanger Institute, Hinxton, United Kingdom; 66 Division of Intramural Research, National Heart, Lung, and Blood Institute (NHLBI), Bethesda, Maryland, United States of America; 67 Human Genetics Center, University of Texas Health Science Center, Houston, Texas, United States of America; 68 Department of Molecular Neuroscience and Reta Lila Laboratories, Institute of Neurology, University College London, London, United Kingdom; 69 Clinical Research Branch, National Institute on Aging, Baltimore, Maryland, United States of America; 70 Departments of Epidemiology, Medicine and Health Services, University of Washington, Seattle, Washington, United States of America; 71 Group Health Research Institute, Group Health, Seattle, Washington, United States of America; 72 Cardiology Division, Massachusetts General Hospital, Harvard Medical School, Boston, Massachusetts, United States of America; 73 Division of Cardiovascular Medicine, Department of Internal Medicine, University of Michigan, Ann Arbor, Michigan, United States of America; Queensland Institute of Medical Research, Australia

## Abstract

White blood cell (WBC) count is a common clinical measure from complete blood count assays, and it varies widely among healthy individuals. Total WBC count and its constituent subtypes have been shown to be moderately heritable, with the heritability estimates varying across cell types. We studied 19,509 subjects from seven cohorts in a discovery analysis, and 11,823 subjects from ten cohorts for replication analyses, to determine genetic factors influencing variability within the normal hematological range for total WBC count and five WBC subtype measures. Cohort specific data was supplied by the CHARGE, HeamGen, and INGI consortia, as well as independent collaborative studies. We identified and replicated ten associations with total WBC count and five WBC subtypes at seven different genomic loci (total WBC count—6p21 in the *HLA* region, 17q21 near *ORMDL3*, and *CSF3*; neutrophil count—17q21; basophil count- 3p21 near *RPN1* and *C3orf27*; lymphocyte count—6p21, 19p13 at *EPS15L1*; monocyte count—2q31 at *ITGA4*, 3q21, 8q24 an intergenic region, 9q31 near *EDG2*), including three previously reported associations and seven novel associations. To investigate functional relationships among variants contributing to variability in the six WBC traits, we utilized gene expression- and pathways-based analyses. We implemented gene-clustering algorithms to evaluate functional connectivity among implicated loci and showed functional relationships across cell types. Gene expression data from whole blood was utilized to show that significant biological consequences can be extracted from our genome-wide analyses, with effect estimates for significant loci from the meta-analyses being highly corellated with the proximal gene expression. In addition, collaborative efforts between the groups contributing to this study and related studies conducted by the COGENT and RIKEN groups allowed for the examination of effect homogeneity for genome-wide significant associations across populations of diverse ancestral backgrounds.

## Author Summary

WBC traits are highly variable, moderately heritable, and commonly assayed as part of clinical complete blood count (CBC) examinations. The counts of constituent cell subtypes comprising the WBC count measure are assayed as part of a standard clinical WBC differential test. In this study we employed meta-analytic techniques and identified ten associations with WBC measures at seven genomic loci in a large sample set of over 31,000 participants. Cohort specific data was supplied by the CHARGE, HeamGen, and INGI consortia, as well as independent collaborative studies. We confirm previous associations of WBC traits with three loci and identified seven novel loci. We also utilize a number of additional analytic methods to infer the functional relatedness of independently implicated loci across WBC phenotypes, as well as investigate direct functional consequences of these loci through analyses of genomic variation affecting the expression of proximal genes in samples of whole blood. In addition, subsequent collaborative efforts with studies of WBC traits in African-American and Japanese cohorts allowed for the investigation of the effects of these genomic variants across populations of diverse continental ancestries.

## Introduction

The WBC count, a classic marker of immune or inflammatory response, varies substantially among healthy individuals. The counts of constituent cell subtypes comprising the WBC count measure are assayed as part of a standard clinical WBC differential test. While the WBC count and WBC differential count are often obtained to assess for evidence of infection or underlying inflammation, prospective epidemiologic studies have consistently linked higher WBC counts, within the clinically designated normal range, along with other inflammatory markers, to increased risk of coronary artery disease, cancer, and total mortality [1], [2], [3], [4], [5], [6], [7], [8], [9], [10]. Studies are often not consistent on the specific WBC subpopulations involved, but granulocytes, in general, and neutrophils, in particular, are most often implicated in these observations [11], [12]. In the general population, the total WBC count is also directly associated with many cardiovascular disease risk factors, such as higher blood pressure, cigarette smoking, adiposity, lower socioeconomic status, and higher levels of plasma inflammatory markers [13].

WBC counts are also moderately heritable [14], with heritability estimates varing from approximately 0.14 to 0.4 across total leukocyte count and cell subtypes, as assessed in a Sardinian population, with the highest heritability estimates for monocyte counts [14]. In addition, the substantially lower neutrophil count and total WBC count in African Americans compared to European-ancestry individuals seems to be at least partially explained by a regulatory variant in the Duffy Antigen Receptor for Chemokine (*DARC*) gene, which accounts for ∼20% of total variation in the measures [15], [16]. Recent studies have sought to investigate the common genetic variants associated with several blood count traits in European-ancestry and Japanese individuals, but have not focused specifically on the multiple cell types comprising the total WBC count measurement [17], [18], [19], [20].

In the current study, we sought to identify and replicate common genetic variants that influence normal variation in six WBC phenotypes commonly measured in the clinical setting and in population studies that comprise the CHARGE Consortium [21], the HaemGen Consortium [18] and independent collaborative studies. We utilized genome-wide association study (GWAS) data and meta-analytic techniques to identify 10 genome-wide significant loci in a study of over 31,000 individuals (Table 1), examining variants possibly associated with total WBC count, three granulocyte phenotypes (neutrophil, basophil and eosinophil counts), and two non-granulocyte phenotypes (lymphocytes and monocytes). We also investigated the shared functional connectivity of identified loci across phenotypes with regards to both known biological pathways and nearby gene expression effects. As previous research has shown strong selective pressures at the locus identified to affect WBC counts in African American populaftions, we examined the possibility of recent selective effects at significantly associated loci in European ancestry individuals [15], [16]. Additional efforts were made in collaboration with RIKEN and COGENT investigators to identify homogenous associations across populations of diverse ancestral backgrounds.

**Table 1:** Descriptive statistics of contributing cohorts.

Discovery Phase							
Study	AGES	ARIC	BLSA	FHS	Health ABC	InChianti	RS
*Total WBC*							
WBC count	6.010 (1.793)	5.933 (1.400)	5.441 (1.099)	4.07 (0.235)	6.166 (1.373)	5.965 (1.260)	6.487 (1.496)
*Granulocytes*							
Basophils	0.029 (0.025)	0.025 (0.033)	0.012 (0.015)	N.A.	0.060 (0.031)	0.026 (0.019)	N.A.
Eosinophils	0.207 (0.147)	0.104 (0.103)	0.174 (0.093)	N.A.	0.173 (0.102)	0.171 (0.091)	N.A.
Neutrophils	3.511 (1.295)	3.646 (1.118)	3.149 (0.868)	N.A.	3.658 (1.010)	3.626 (1.020)	N.A.
*Non-granulocytes*							
Lymphocytes	1.726 (0.943)	1.814 (0.479)	1.636 (0.445)	N.A.	1.741 (0.580)	1.834 (0.527)	2.500 (0.781)
Monocytes	0.538 (0.177)	0.344 (0.139)	0.406 (0.145)	N.A.	0.534 (0.150)	0.310 (0.091)	N.A.
Covariates							
% Female	58.0	53.2	48.7	51.2	47.1	57.0	59.5
% Current smoker	12.7	21.1	2.7	42.2	4.3	17.7	22.6
Age in years	76.4 (5.5)	54.3 (5.7)	66.8 (13.9)	35.9 (10.4)	75.7 (2.8)	68.1 (15.3)	69.1 (9.0)
Sample Sizes							
Total N	3217	4846	337	3909	1075	1014	5111

Replication Phase										
Study	HVH	INGI-CARL	INGI-FVG	INGI-VB	KORAF3	KORAF4	RSII	SORBS	TwinsUK	UKBS1
*Total WBC*										
WBC count	6.932 (2.076)	7.176 (2.900)	5.867 (1.414)	6.250 (1.242)	6.781 (1.671)	5.904 (1.638)	6.468 (1.388)	5.204 (1.159)	5.926 (1.530)	7.255 (1.464)
*Granulocytes*										
Basophils	0.068 (0.034)	N.A.	N.A.	0.029 (0.014)	N.A.	N.A.	N.A.	0.027 (0.014)	N.A.	0.100 (0.001)
Eosinophils	0.204 (0.158)	N.A.	N.A.	0.185 (0.100)	N.A.	N.A.	N.A.	0.150 ( 0.082)	N.A.	0.182 (0.092)
Neutrophils	4.193 (1.586)	N.A.	N.A.	3.444 (0.917)	N.A.	N.A.	N.A.	2.853 (0.837)	N.A.	4.268 (1.105)
*Non-granulocytes*										
Lymphocytes	1.932 (0.799)	2.492 (0.682)	2.083 (0.141)	2.141 (0.549)	N.A.	N.A.	2.236 (0.568)	1.749 (0.531)	N.A.	2.293 (0.593)
Monocytes	0.534 (0.189)	0.415 (0.346)	0.786 (0.232)	0.451 (0.126)	N.A.	N.A.	0.455 (0.164)	0.424 (0.119)	N.A.	0.446 (0.129)
Covariates										
% Female	53.0	58.2	59.4	57.0	50.6	51.2	57.2	40.1	100.0	52.0
% Current smoker	8.4	25.5	21.7	14.1	13.3	14.7	16.1	12.9	N.A.	N.A.
Age in years	68.1 (9.1)	44.2 (20.0)	48.3 (19.3)	54.8 (18.0)	62.5 (10.1)	60.9 (8.9)	67.8 (7.1)	47.9 (16.0)	50.9 (13.4)	43.4 (12.4)
Sample Sizes										
Total N	403	510	1045	1405	1640	1813	1518	785	1466	1238

The numbers above are thousands of cells per milliliter of blood. All values are presented as mean (SD), except where % indicated.

## Results

**Figure 1:**
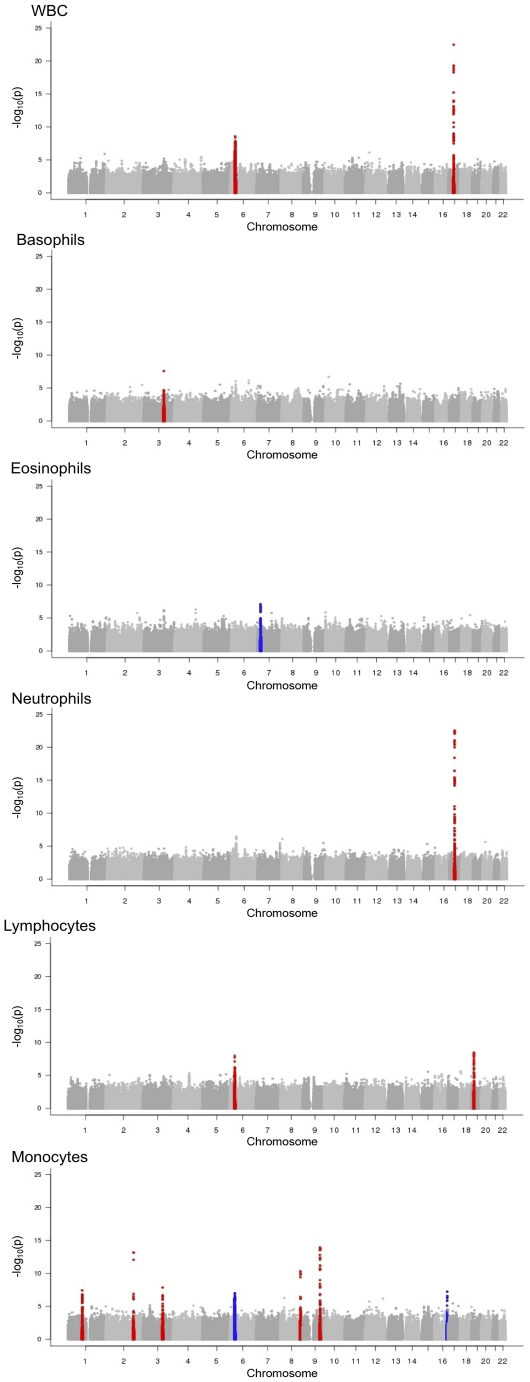
Results of discovery phase meta-analyses for white blood cell phenotypes. Red regions denote loci containing SNPs reaching genome-wide significance at p-values<5.00E-08. Blue regions denote suggestive loci containing SNPs with p-values between 5.00E-08 and 1.00E-07.

In the discovery meta-analysis of 19,509 subjects from seven cohorts, we identified 11 genome-wide significant associations with six white cell phenotypes (total WBC, neutrophil, basophil, eosinophil, lymphocyte and monocyte counts, see Table 1, Table S1, Figure 1). Further, we found strong evidence for replication of 10 of the 11 trait-locus associations in 11,823 independent samples from 10 GWAS cohorts who contributed summary statistics for SNPs of interest. The discovery analysis results (Figure 1 and Figures S1, S2, S3, S4, S5, S6, S7, S8, S9, S10 for details), and the results of replication testing for the 10 replicated trait-loci are summarized in Table 2. These results are presented in greater detail for all genome-wide significant SNPs in Table S2.

**Table 2:** Summary results from discovery and replication phase analyses for all genome-wide significant and successfully replicated loci.

				Discovery Phase			Replication Phase			Joint Estimates								
Phenotype	SNP	Effect Allele	Alternative Allele	Beta	SE	P-value	Beta	SE	P-value	Beta	SE	P-value	Effect Heterogeneity P-value	CHR	Cytoband	BP	Genes +/−100 kb from most significant SNP in locus	Variance explained (adjusted r2) per trait
**WBC**	rs2517524	a	c	0.017	0.003	2.64E-09	0.011	0.003	2.50E-05	0.013	0.002	1.46E-12	0.443	6	6p21.33	31133692	*MUC21, HCG22, C6orf15, CDSN, PSORS1C1, PSORS1C2 and CCHCR1*	0.070
	rs4794822	t	c	0.028	0.003	3.23E-23	0.018	0.002	1.31E-14	0.022	0.002	1.72E-34	0.053	17	17q21.1	35410238	*GSDMB, ORMDL3, GSDMA, PSMD3, CSF3, MED24, SNORD124, THRA and NR1D1*	
**Neutrophils**	rs8078723	t	c	−0.043	0.004	2.84E-23	−0.036	0.006	4.99E-10	−0.041	0.004	3.16E-31	0.358	17	17q21.1	35420405	*GSDMB, ORMDL3, GSDMA, PSMD3, CSF3, MED24, SNORD124, THRA and NR1D1*	0.067
**Basophils**	rs4328821	a	g	0.010	0.002	2.58E-08	0.010	0.002	8.40E-06	0.010	0.001	8.72E-13	0.218	3	3q21.3	129799125	*LOC90246, C3orf27 and RPN1*	0.021
**Lymphocytes**	rs2524079	a	g	0.014	0.003	1.85E-08	0.018	0.005	2.03E-04	0.015	0.002	2.93E-11	0.667	6	6p21.33	31348700	*PSORS1C3, HCG27, HLA-C and HLA-B*	0.058
	rs11878602	a	c	−0.016	0.003	3.42E-09	−0.014	0.004	9.69E-04	−0.015	0.002	8.43E-12	0.975	19	19p13.11	16416153	*EPS15L1, CALR3, C19orf44 and CHERP*	
**Monocytes**	rs1449263	t	c	0.036	0.005	6.71E-14	0.037	0.006	2.13E-10	0.036	0.004	5.21E-23	0.999	2	2q31.3	182027546	*ITGA4, CERKL*	0.088
	rs9880192	c	g	−0.028	0.005	1.35E-08	−0.027	0.006	2.08E-05	−0.028	0.004	1.23E-12	0.888	3	3q21.3	129780259	*GATA2, LOC90246, C3orf27 and RPN1*	
	rs1991866	c	g	−0.032	0.005	4.58E-11	−0.037	0.006	7.51E-10	−0.034	0.004	1.79E-19	0.747	8	8q24.21	130693287	*NA*	
	rs10980800	t	c	−0.044	0.006	1.13E-14	−0.039	0.007	7.03E-09	−0.042	0.004	4.26E-22	0.625	9	9q31.3	112955726	*EDG2*	

SNPs included are the most significant SNP per genomic region of interest to aid in clarity.

Total WBC count was associated with two independent loci in the discovery phase of analyses; the first locus was on chromosome 6p21 encompassing a region from 31,131,127 bp to 31,161,846 bp near *HLA* and *PSORS1* gene families, and the second locus on chromosome 17q21 from 35,345,186 bp to 35,470,048 bp near candidate genes *ORMDL3* and *CSF3*. Both loci showed independent replication (Table 2). Neutrophil count was associated with a 196,381 bp region on chromosme 17q21 containing 46 genome-wide significant SNPs from the discovery phase. This region overlaps the locus on chromosome 17q21 identified for the total WBC count phenotype and showed positive association in replication testing at all but 2 SNPs. Basophil count was associated with one SNP, rs4328821 on chromosome 3q21 near *RPN1* and *C3orf27*. This SNP showed a significant positive association between basophil count and minor allele dosage (minor allele frequency 0.110, p-value in discovery phase 2.58E-08, p-value in replication phase 8.40E-06). No regions showed genome-wide significance for association with eosinophil count.

Lymphocyte count was associated with two loci, with one locus on chromosome 6p21 overlapping with the chromosome 6p21 total WBC count locus. The second locus associated with lymphocyte counts is on chromosome 19p13, from 16,32,871 bp to 16,429,197 bp at *EPS15L1* and was successfully replicated. Monocyte count was associated with the largest number of independent hits for any of the traits, with five loci identified in the discovery analysis, four of which showed significant associations in replication testing. These four loci include two intergenic regions (>100 kb to nearest known genes) on chromosome 8q24 (from 130,672,817 to 130,693,287) and chromosome 9q31 (112,917,232 to 113,073,157). We also identified a novel association on chromosome 2q31 (182,027,546 to 182,036,459) near *ITGA4*, and a single genome-wide significant SNP on chromosome 3q21, which is located 18,866 bp from the SNP significantly associated with basophil count (r^2^ = 0.076, D′ = 0.841). The monocyte- associated locus on chromosome 1q22 contained only one genome-wide significant SNP which failed to replicate and is not included in Table 2 (denoted by rs17131683, which exhibited a replication p-value of 0.770 but a consistent negative effect associated with the A allele).

Many of the loci described in this report contain genome-wide significant SNPs spread throughout much of each locus suggestive of either extensive LD or multiple association signals at each locus. For loci described in Table S2 (except those containing less than 3 genome-wide significant SNPs), conditional analyses were conducted using the allele dosage of the most significant SNP per locus as a covariate in a subset of discovery cohorts (AGES, ARIC, BLSA, Health ABC and InChianti). Statistical models were identical to those used in the discovery analyses except for the additional SNP covariates. No SNPs analyzed remained significant after correcting for 147 tests, showing that only one signal of association exists per locus. The complete results for these analyses are evident in Table S3. As each locus accounts for only one unique signal per trait, adjusted r^2^ estimates were calculated for each trait across loci within this subset of cohorts, and may be found in Table 2.

To assess the independence of associated SNPs from the total WBC count measurement, all genome-wide significant SNP associations for white cell subtypes (Table S2) were re-analyzed as per the discovery phase analysis methods adjusting for total WBC count as an additional covariate in a subset of discovery cohorts (AGES, ARIC, BLSA, Health ABC and InChianti). After Bonferroni correction for 97 independent tests at least one SNP per locus remained significant, suggesting some independence from the total WBC measure in the SNP associations. The extended results of this analysis and a table of r^2^ values for the phenotypes of interest based on the same subset of discovery cohorts may be found in Table S4 and Table S5.

These results demonstrate a high degree of relatedness across traits, with individual loci affecting multiple WBC traits, that may be pleiotropic to some degree or due to the biological relatedness of the traits. Neutrophils are the most abundant WBC subtype, and the locus on chromosome 17q21 associated with both total WBC count and neutrophil count independently based on conditional analyses described above. In this region, 38 SNPs were common to both traits in the discovery analysis, and these SNPs showed identical directions of effects across both traits. In the chromosome 3q21 between *C3orf27* and *RPN1*, rs9880192 was associated with monocyte count and rs4328821 was associated with basophil count, suggesting pleiotropy in this region. The region on chromosome 6p21 near the *PSORS1* family of genes as well as *HLA-C* and *HLA-B* contains associated SNPs with both total WBC count and lymphocyte count, although, interestingly, spatially overlapping SNPs failed to replicate in the other trait, further suggesting independence of effects seen in conditional analyses of all loci.

**Figure 2:**
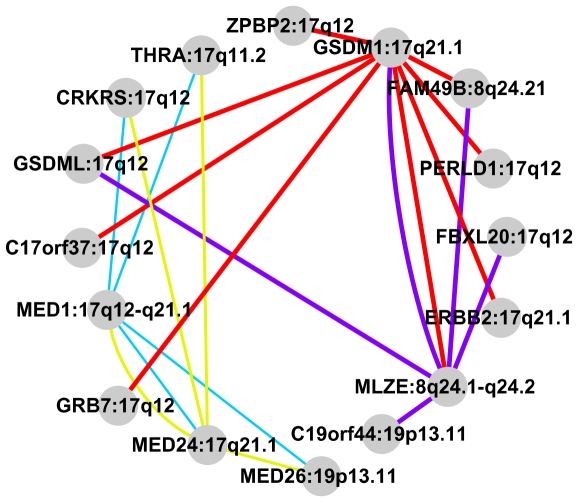
GRAIL gene clusters represented graphically for all clusters reaching a FDR adjusted p-value <0.05. The relative thickness of each line connecting nodes is indicative of a p-value closer to zero. Gene symbols are designated on each node, with the cytogenetic position following the colon. Individual gene clusters are color-coded.

To further examine the functional connectivity of these loci across white blood cell phenotypes, the Gene Relationships Among Implicated Loci package (GRAIL, http://www.broad.mit.edu/mpg/grail/) was utilized to mine PubMed archives using textual analyses of known functional associations to identify concurrent effects and relationships across phenotypes [22]. In brief, GRAIL incorporates functional annotations from text mining related to specific genomic loci, usually genome-wide association study results, to assess the functional inter-relatedness of genes in linkage disequilibrium with the regions of interest and construct networks of related genes sharing biological function. In our analyses, we utilized GRAIL to survey the functional relatedness of all regions containing significant results passing Bonferroni correction in both the discovery and replication phases. We identified four clusters of related genes with false-discovery rate adjusted p-values<0.05 out of the 49 gene clusters generated by the GRAIL package, which are described in Figure 2 and Table S6. All four clusters show significant interconnectivity between genes proximal to loci on chromosome 17q21 associated with total WBC and neutrophil counts and the chromosome 19p13 locus associated with lymphocyte count. The two most significant clusters also show relationships between genes proximal to the previously mentioned loci and candidate genes at the chromosome 8q24 region associated with monocyte counts. Genes at the chromosome 17q21 locus associated with both total WBC and neutrophil counts appeared in all significant pathways identified in the GRAIL analyses, suggesting biological connectivity across both granulocyte and non-granulocyte cell lineages. Candidate genes from the gasdermin (*GSDML*) and mediator complex subunit (*MED*) families were highly enriched in the significant gene clusters and comprised 37.5% of genes in these clusters. These results suggest shared biological pathways between these genes and cell proliferation among WBC subtypes associated with these genomic regions, although as emphasized in conditional analyses, these effects at these loci remain to some degree independent of the total WBC measure.

**Figure 3:**
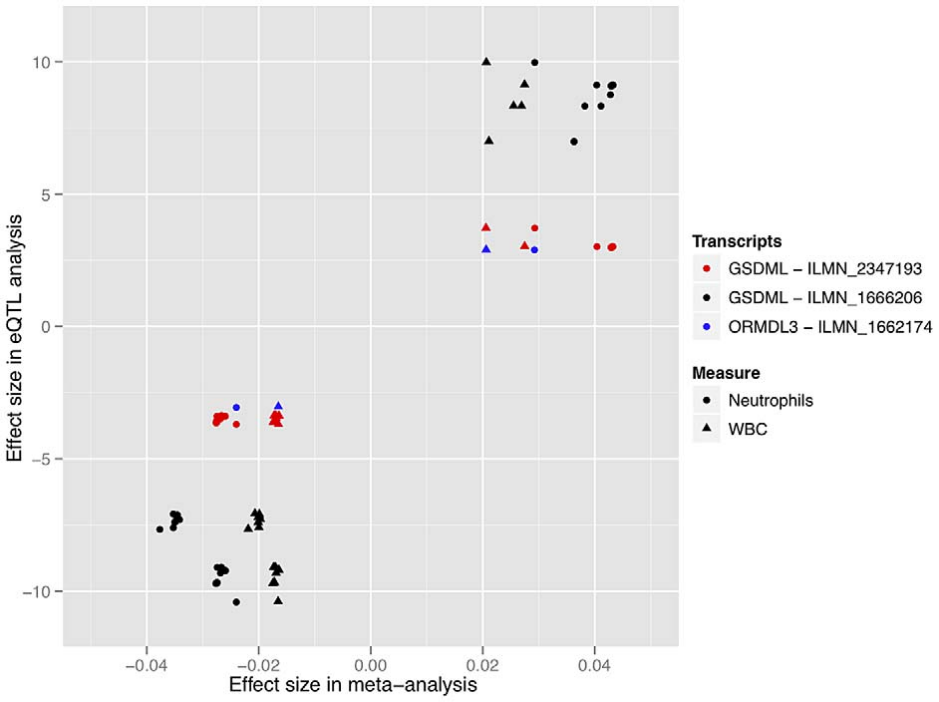
The directionality and magnitude of effects for SNPs significant in both the meta-analysis and eQTL analysis are highly correlated (r^2^ = 0.795). Effect estimates in both the eQTL analysis and meta-analysis were standardized to effects based on dosages of the minor allele for each SNP. Meta-analysis effects are based on beta coefficients from regression, while eQTL effects are based on standardized betas. This figure only includes significant SNPs to improve clarity.

We also examined possible functional consequences of individual SNP associations by analyzing whole blood genome-wide gene expression data from the InChianti study to identify associations between SNPs found to be significant in the meta-analysis and cis changes in gene expression. All SNPs significant in both the discovery and replication phases for all phenotypes were used in the expression analysis. Each SNP in this dataset which was within 500 kb of an expression probe was treated as a possible expression quantitative trait locus (eQTL). For 85 SNPs in our subset of significant meta-analysis results, we tested at total 741 candidate eQTL associations using multivariate linear regression. This analysis tested each SNP in the subset for an association with each expression probe within 500 kb. After Bonferroni correction for the 741 tests conducted, 36 SNPs in the chromosome 17q21 locus associated with total WBC count and neutrophil count in the meta-analysis were also significantly associated with cis-expression levels. In total, these 36 SNPs were associated with three expression probes, 2 probes tagging transcripts in the *GSDML* gene (probes ILMN_2347193 and ILMN_1666206) and another probe tagging a single probe in *ORMD3L* (probe ILMN_1662174), for a total of 103 signficicant eQTLs (Figure 3). Both probes in *GSDML* were highly correlated (r^2^ = 0.582), although neither *GSDML* probe was strongly corellated with the probe of interest in the *ORMD3L* gene (r^2^<0.200). With each SNP's minor allele as the point of reference, all effect directions for significant meta-analysis and eQTL associations were concordant, showing a strong correlation between the effect sizes in the meta-analysis and eQTL analysis, suggesting that the effect of the identified SNPs on the corresponding WBC trait may be transcriptionally mediated. For example, the correlation of effect estimates between the eQTL and meta-analysis for neutrophil associated SNPs also associated with the ILMN_1666206 probe highly significant, with an r^2^ of 0.898. For details of all eQTLs tested, please refer to Table S7 and the Methods and Materials section.

Previous studies of WBC genetic variation in African American populations have shown evidence of WBC associated loci in a region of high selective pressures, with the putative functional SNP rs2814778 being almost fixed in frequency in sub-Saharan African populations where malaria is endemic [15]. We therefore undertook an investigation of recent selective pressures acting upon SNPs identified associated with WBC phenotypes in European-ancestry populations. Evidence for selection for all 10 significant trait-loci from the meta-analysis was evaluated by mining HapMap2 CEU data. Integrated Haplotype Scores (iHS) were used to quantify selection at each locus based on homozygosity decay in extended haplotypes and are availible for download from the Haplotter website (http://hg-wen.uchicago.edu/selection) [23]. Selection was quantified by an absolute value of iHS>2 indicating strong selective pressures, and absolute iHS values ≤2 and ≥1.635 were interpreted as indicating recent-moderate selection, positive or negative iHS values indicated the direction of selection [24]. All replicated SNPs were evaluated for signatures of selection. One locus showed evidence of selection, and this was the lymphocyte associated locus on chromosome 19p13, from 16,336,388–16,429,197 bp. This locus on chromosome 19p13 showed evidence of selection for all SNPs analyzed in the replication phase. All SNPs in this locus appear to be under some degree of negative selection with 2 of the 11 SNPs being under strong negative selection and the rest under recent moderate selection. However, when testing the correlation between effect estimates and iHS at this locus using the ancestral allele as a reference for effect direction, no clear correlation between iHS and effect size was detected (r^2^ = 0.226, p-value = 0.140). Full iHS annotation for replicated SNPs are shown in in Table S8.

As part of collaborative efforts with the COGENT and RIKEN groups, a coordinated exchange of summary statistics for SNP identified in Table 2 was organized from the parallel studies conducted by these groups. Random-effects meta-analysis techniques were utilized to identify effects that were consistent across studies of diverese ethnic backgrounds. While all joint effect estimates remained in consistent directions with those described in Table 2, heterogeneity of effects across the 3 ancestral populatons severely attenuated the strength of the associations for all but 2 of the genome-wide significant associations identified in this report. The associations at rs4794822 (WBC count) and rs11878602 (lymphocyte count) remained genome-wide significant. Consistent robust effects across ancestral populations at rs11878602 may lend some support to the recent-moderate negative selection at this SNP (iHS = −1.924) being associated with an increase in frequency of the derived A allele associated with decreasing monocyte counts. These results suggest that these SNPs may be very close to the functional variants associated with these effects, as well as exhibiting relatively consistent effects across multiple population ancestries with differing LD structures. The results of these analyses are detailed in Table 3.

**Table 3:** Results of random-effects meta-analyses incorperating summary statistics from COGENT, RIKEN, and CHARGE meta-analyses for loci of interest.

		European Ancestry - CHARGE and HeamGen			African American Ancestry - COGENT Network			Japanese Ancestry - Riken Institute			Random Effects Meta-analysis				
Phenotype	SNP	Beta	SE	P-value	Beta	SE	P-value	Beta	SE	P-value	Summary Effect	Summary SE	P-value	Heterogeneity Variance	Heterogeneity P-value
**WBC**	rs2517524	0.013	0.002	1.46E-12	0.007	0.004	0.058	0.002	0.003	0.437	0.008	0.004	0.029	3.19E-05	0.008
	rs4794822	0.022	0.002	1.72E-34	0.013	0.004	7.56E-04	0.019	0.003	1.25E-12	0.019	0.002	3.44E-15	9.74E-06	0.088
**Neutrophils**	rs8078723	−0.041	0.004	3.16E-31	−0.007	0.007	0.331	−0.034	0.005	5.60E-11	−0.028	0.009	1.19E-03	1.99E-04	1.82E-04
**Basophils**	rs4328821	0.010	0.001	8.72E-13	0.002	0.001	0.046	0.009	0.001	4.67E-25	0.007	0.003	6.37E-03	1.82E-05	3.11E-08
**Lymphocytes**	rs2524079	0.015	0.002	2.93E-11	0.008	0.005	0.097	0.003	0.004	0.394	0.009	0.004	0.025	3.63E-05	0.015
	rs11878602	−0.015	0.002	8.43E-12	−0.015	0.005	2.20E-03	−0.008	0.003	0.016	−0.013	0.002	1.84E-08	5.53E-06	0.224
**Monocytes**	rs1449263	0.036	0.004	5.21E-23	0.009	0.003	5.01E-03	0.041	0.006	2.92E-12	0.028	0.011	9.13E-03	3.34E-04	3.69E-10
	rs9880192	−0.028	0.004	1.23E-12	0.004	0.005	0.431	−0.038	0.011	6.74E-04	−0.020	0.013	0.125	4.43E-04	2.01E-07
	rs1991866	−0.034	0.004	1.79E-19	−0.005	0.003	0.138	−0.025	0.006	2.92E-05	−0.021	0.010	0.041	2.98E-04	4.73E-09
	rs10980800	−0.042	0.004	4.26E-22	−0.005	0.004	0.185	0.014	0.013	0.263	−0.012	0.016	0.428	6.75E-04	1.42E-11

## Discussion

This meta-analysis has identified ten genome-wide significant trait-locus associations with WBC phenotypes spread across seven genomic loci. Of these trait-locus associations, seven associations represent novel findings, and three associations, across two genomic loci, confirm previously identified associations. The association of chromosome 17q21 near *ORMDL3* and *CSF3* associated in our study with total WBC count and neutrophil count, and the 9q31 locus associated with monocyte count have been previously demonstrated in European-ancestry and Japanese populations [25], [26]. The chromosome 3q21 locus near *RPN1* and *C3orf27* has been previously shown to be associated with eosinophil count, and is instead associated with related granulocyte cell measures of monocyte and basophil counts in this study, although we do identify suggestive p-values at ∼1.00E-4 to 1.00E-8 and the same direction of effect for the additional loci identified in Gubjartsson et al., 2010 [19], [27]. In addition, a number of previous GWAS have implicated the monocyte count associated locus at chromosome 8q24.21 as affecting height, renal function, serum protein levels, multiple sclerosis, glioma, leukemia and a number of other cancers [28]–[59]. Through conditional analyses and an analysis of functional relatedness, we have shown correlation among related traits and possible pleiotropic connectivity of these loci across phenotypes that represent measures of biologically related cellular lineages, as well as identified loci showing a direct association between allelic gene expression differences and variation in phenotypic measures.

The associations identified in this study are robust, and several have been previously identified in GWAS studies of immunologically relevant phenotypes, such as the association of celiac disease with the chromosome 2q31.1 locus containing *ITGA4*. *ITGA4* encodes an alpha integrin subunit present on monocytes, lymphocytes, endothelial cells and erythrocytes that serves as an adhesion molecular receptor for VCAM-1, fibronectin. VCAM-1 is expressed at high levels on the vasculature of the bone marrow, and therefore alpha4integrin receptors may play a role in homing and recruitment of certain cell types during hematopoiesis [60], [61].

The overlapping loci on chromosome 17q21 associated with both total WBC and neutrophil counts constitute a single plausible candidate locus contributing to general variability in total WBC count and neutrophil count via hematopoietic mechanisms. These measures are highly correlated, and the estimated SNP effects are also likely correlated for this reason. From a functional perspective, the role of G-CSF, the *CSF3* gene product, has been well-described in the biology of myeloid progenitor production and differentiation. This locus previously reached genome-wide significance in a joint analysis of discovery and replication cohorts of total WBC count in individuals of European ancestry [18]. The same locus, containing the genes *PSMD3* and *CSF3*, was associated with neutrophil count in a cohort of Japanese participants [27]. This study of Japanese subjects also reported a significant genome-wide association with neutrophil count for three SNPs in a locus at chromosome 20, containing the gene *PLCB4*. Due to the lower minor allele frequency in European ancestry individuals of these three correlated SNPs (minor allele frequency = 0.076 in HapMap CEU samples versus 0.289 in HapMap JPT samples for rs2072910) and the marginal effect size detected in the original report, statistical power to detect this association was limited in our analysis [27].

Our data suggest that the locus on chromosome 17q21 has functional connectivity across white cell subtypes. Multiple genes at this locus appeared in all significant pathways identified in the GRAIL analyses showing a functional connectivity across both granulocyte and non-granulocyte cell lineages. Gene clusters detailed in the GRAIL analyses show significant functional clusters including genomic regions that are separately associated with granulocyte and non-granulocyte traits within the same cluster. However, the results of the GRAIL analyses may be influenced by both funding avenues and publication bias, as the classifications are based only on PubMed searchable published results.

Cis-eQTL effects on transcripts in the *ORMDL3* and *GSDML* were shown to be highly correlated with allelic effects associated with neutrophil and total WBC count measures quantified in the meta-analysis. This suggests that variation in total WBC count and neutrophil counts are at least in part due to polymorphism-based regulation of gene expression. This regulation of gene expression by SNPs in this region may be related to some form of systemic immunological function, as variants in this region were also shown to be associated with expression of *ORMDL3* in childhood asthma [62]. Interestingly, Okada et al. also showed significant eQTL associations between SNPs and transcripts at this locus, although their associations implicate regulation of *PSMD3* as affecting neutrophil variation, rather than the exact transcript associations identified in this report [27]. This slight difference may be attributable to the larger sample size in our study, differences in population ancestry of the two studies, and the use of expression data derived from lymphoblastoid cell lines instead of whole blood. The *CSF3* gene at this locus is a possible candidate contributing to effects across multiple cell subtypes at this locus, as functional studies have demonstrated that this gene creates a protein integral to the differentiation and functionality of granulocyte cells [63], [64], [65], [66]. One important caveat to our eQTL analysis is the lack of representation of the *CSF3* gene on the expression array used.

The loci on chromosome 6p21 associated with total WBC and lymphocyte counts appear to be independent of each other as the lymphocyte association persists after adjustment for total WBC. While the closest replicated SNP associations are roughly 200 kb from each other, and a shared functional connectivity between these regions was not elucidated in the GRAIL analysis. At this locus, rs2524079 associated with lymphocyte count is in moderate LD with a number of SNPs in the periphery of the total WBC count locus, including rs2844619, a SNP significant in the total WBC count discovery phase analysis (D′ = 0.762, r^2^ = 0.305, from HapMap Phase II CEU samples). The finding of multiple independent effects at a single locus has occurred in prior studies and include examples such as the finding of two independent signals within the *PLAG1* locus associated with human height, suggesting a locus specific effect, in the current example affecting leucopoiesis or leukocyte homeostasis [48]. Our chromosome 6p21 WBC and lymphocyte loci, both harbor candidate genes that have been previously implicated as associated with phenotypes closely related to immunological function. The locus associated with total WBC count on chromosome 6p21 contains genes associated with follicular lymphoma (*CHCG22*), progression of HIV-1 infection (*CDSN*, *PSORS1C1* and *PSORS1C2*) and psoriasis (*CCHCR1*, *PSORS1C1* and *PSORS1C2*) [67], [68], [69]. This gene rich region includes *HLA* family genes, particularly *HLA-B* and *HLA-C* that are candidates within the lymphocyte associated locus, and actually overlap with the psoriasis candidate locus identified previously via linkage mapping studies and includes *PSORS1C1* and *PSORS1C2*, showing the relatedness of these two loci on chromosome 6p21 [69], although conditional analyses adjusting for total white blood cell count validate these as primarily independent effects. The lymphocyte associated regions containing *HLA-B* and *HLA-C*, harbors two genes that have been implicated in multiple GWAS as modifiers of immunological responses, associated with IL-18 levels, HIV-1 control, vitiligo, multiple clerosis, and psoriasis [49], [68], [70], [71], [72], [73], [74], [75], [76].

The region associated with basophil and monocyte counts on chromosome 3q21 proximal to the *GATA2* gene was previously described as associated with variation in eosinophil count in European and Asian ancestry populations [19]. This association with eosinophil count was not identified as genome-wide significant in our analyses, with multiple SNPs in the GATA2 region (GATA2+/−250 kb) approaching genome-wide significance, exhibiting p-values in the discovery meta-analysis including a regional minimum of 6.73E-07 (rs4328821 ). This is likely due to our decreased sample size for analyses of eosinophil count compared to the original report. However, our data show a significant association between this genomic region and basophil count, and basophils and eosinophils are both granulocytic cells with common lineage in WBC differentiation. GATA2 is a well-known transcription factor involved in maintenance of early hematopoietic cell pools and proximal hematopoietic pathways. In addition, this region proximal to *GATA2* is also significantly associated with monocyte counts, showing overlapping associations across both granulocyte and non-granulocyte cell lineages and supporting the previously described functional role of *GATA2* more proximally in the WBC differentiation process [77], [78]. The independence across granulocyte and non-granulocyte lineages is evident as both associations showed independent signals of association after adjustment for total WBC.

Our analyses have identified genomic loci associated with total WBC and constituent white cell subtypes in European ancestry cohorts. Our findings differ from the results of similar studies of African American and Asian ancestry populations. Population variation at previously identified total WBC count associated loci of *DARC* in African American cohorts and *PLCB4* in Asian ancestry samples motivated our investigation of possible selection at the loci identified in this report, as allele frequencies of SNPs in *DARC* and *PLCB4* differ across populations. This may be suggestive of recent selection. Our analyses of iHS statistics for genome-wide significant SNPs yielded only one locus under selection, with all SNPs investigated in this region being under negative selection in European ancestry populations. These SNPs on chromosome 19p13 associated with lymphocyte counts are proximal to candidate genes such as *CHREP*, which function in calcium homeostasis in lymphocytes, and mutations in the coding region of *CALR3* are associated with familial hypertrophic cardiomyopathy [79], [80]. A thorough search of literature did not reveal any known selective factors associated with this locus. In addition, the fact that this locus remained genome-wide significant in random-effects modeling across diverse ancestral populations suggests a highly generalizable effect at this locus that may or may not be related to selective factors.

In conclusion, we have identified and replicated a set of 10 independent trait-locus associations influencing multiple related WBC traits, of which seven are novel associations. Integrative analyses of our association data and gene expression analyses support pleiotropic effects that will require further functional testing to clarify.

## Materials and Methods

### Ethics Statement

All participating studies conducted their research in accordance with their respective institutional scientific and ethical review boards. All human participants provided informed consent and all clinical investigation was conducted in accordance with the Declaration of Helsinki.

### Study Methods

WBC counts were measured in 19,509 subjects in 7 discovery cohorts (The Rotterdam Study (RS), Framingham Heart Study(FHS), the NHLBI's Atherosclerosis Risk in Communities (ARIC) Study, the Age, Gene/Environment Susceptibility – Reykjavik Study (AGES) Study, Health Aging and Body Composition Study (Health ABC), the Baltimore Longitudinal Study of Aging (BLSA), and the Invecchiare in Chianti Study (InChianti)) and 11,823 subjects in 10 replication cohorts (the Sorbs, the Twins UK cohort (TwinsUK), Kooperative Gesundheitsforschung in der Region Augsburg (KORAF3 & KORAF4) and UK Blood Services Donor Panel 1 (UKBS1) studies, three of the Italian Network on Genetic Isolates (INGI) studies (Carlantino, Val Borbera and Friuli Venezia Giulia), the Rotterdam Study II (RSII) and the Heart and Vascular Health Study (HVH)). In order to study genetic factors affecting variation of these traits within normal ranges, each study excluded all participants with any WBC measure (total WBC or one of the 5 cell subtypes) outside of +/−2 standard deviations from the mean value for that trait.

WBC phenotypes were derived from data provided by fluorescence activated cell sorting technologies commonly employed in clinical and epidemiological studies to interrogate common hematological elements found in peripheral blood. Total WBC count was reported in thousands of cells per ml, and sub-type specific cell counts were calculated by multiplying the proportion of the WBC count comprised by each cell type by the total WBC measure. Any subject with a trait value greater than 2 SD from the corresponding mean of that trait in each cohort or missing data for any assayed phenotype were excluded from all analyses. Shapiro-Wilk tests of normality were implemented in the smallest discovery cohorts as the data was available at the time of study design (the InChianti study and the Baltimore Longitudinal Study of Aging, BLSA) to evaluate normality of the phenotypes for analyses. Raw values, natural log transformed and square-root transformed values for each phenotype of interest in these two studies were compared with regard to deviations from normality based assessment of the Shapiro-Wilk test statistic in the two studies. Based upon these reviews, a uniform analysis plan was established for conducting each study-level analyses, analysis, using either log transformation (total WBC count, neutrophil count, and monocyte count) or square-root transformation (basophil count, eosinophil count and lymphocyte count) in order to normalize the distributions of the phenotypic data.

At the study level, GWAS analyses were conducted on unrelated participants (except for FHS) of confirmed European ancestry based on either multi-dimensional scaling or principal components analyses, concordance between genotypic and self-reported gender, and successfully genotyped at >95% of attempted SNPs. SNPs were filtered based on criteria of minor allele frequency >0.01 (MAF), missingness per SNP<5% and Hardy-Weinberg equilibrium p-value>1.00E-7 (HWE, used to exclude poorly clustered genotypes). Participants and SNPs passing basic quality control were imputed to >2.4 million SNPs based on HapMapII haplotype data. All studies utilized multivariate linear regression to generate study level summary statistics for each phenotype, with allelic dosages at each SNP used as the independent variable and primary covariates of age at hematology assay, current smoking status and sex. Detailed descriptions of participating studies, their quality control practices and study-level analyses which may differ slightly from those described above are provided in Text S1.

To conduct meta-analyses, all studies submitted summary statistics from the study-level linear regression analyses for each phenotype. Meta-analyses were conducted using inverse-variance weighted fixed-effects models to combine beta coefficients and standard errors from study level regression results for each SNP to derive a combined p-value. Prior to discovery meta-analyses, SNPs were excluded if imputation quality metrics (equivalent to the squared correlation between proximal imputed and genotyped SNPs) were less than 0.30. Study level results were also corrected for genomic inflation factors (λ_GC_) by incorporating study specific λ_GC_ estimates into the scaling of the standard errors (SE) of the regression coefficients by multiplying the SE by the square-root of the genomic inflation factor (see Table S1 for study and phenotype specific genomic inflation factors) [81]. Study specific genomic inflation factor estimates for all discovery cohorts were all <1.05 except for 1.12 in the Health ABC analysis of basophil count and 1.09 in the analysis of total white blood cell count in AGES (Table S1). No definitive cause of this inflation could be identified, and of particular interest, the genomic inflation factors for related traits in these two studies were within the normally accepted range. Meta-analyses were implemented using METAL and independently re-analyzed using R to confirm results [82].

We chose *a priori* to carry over all results from discovery meta-analyses at p-values<5.00E-08 to replication meta-analyses, excluding any SNPs with Cochran's Q test of heterogeneity p-values<0.01 or missing in more than 2 studies. These conservative exclusion criteria caused the exclusion of 6 of 167 genome-wide significant SNPs from replication analyses, and these SNPs did not constitute any new loci of interest. The final number of SNPs for replication analyses was then reduced to 161 candidate SNPs across all phenotypes. For replication meta-analyses of individual SNPs, each phenotype was analyzed separately using similar inverse-variance weighted meta-analyses as in the discovery stage analyses, although no genomic control was used. P-values for significant associations in the replication stage were corrected for the number of SNPs tested per phenotype using the standard Bonferroni correction for multiple testing (total WBC count corrected for 63 SNPs, with a significance threshold of p-value≤7.94E-4; neutrophil count corrected for 46 SNPs, with a significance threshold of p-value≤1.09E-3; basophil count corrected for 1 SNP, with a significance threshold of p-value≤0.05; lymphocyte count corrected for 14 SNPs, with a significance threshold of p-value≤3.57E-3; and monocyte count corrected for 37 SNPs, with a significance threshold of p-value≤1.35E-3). Of the 161 candidate SNPs included in the replication phase, 152 SNPs passed the trait-specific replication p-value thresholds. Ony one genome-wide significant locus failed to replicate, and this locus on chromosome 1q22 contained only one genome-wide significant SNP associated with monocyte count in the discovery phase.

Of the 152 successfully replicated associations, 109 SNPs were unique, since some SNPs were significant across multiple phenotypes. These replicated SNPs were analyzed in GRAIL to infer a possible biological connection between significant meta-analysis loci. GRAIL was used to mine textual data based on PubMed keywords to examine functional relatedness across phenotypes based on inferred biological interconectivity between genes proximal to meta-analysis results. SNP (rs) identifiers for these associated SNPs were input into the GRAIL webserver as a means of specifying genomic regions of interest in constructing query and seed regions to be analyzed. Genes for text mining of the functional datasource were identified using the LD structure of HapMap2 Release 22 CEU samples, gathering gene identifiers to search indexed abstracts from PubMed last curated on May 2010. Genes in regions of interest were clustered based on keyword similarity. These genes and clusters were then scored based on ranked similarity, adjusting for gene size, to generate p-values evaluating the strength of the functional interconnectivity of genes in the regions of interest. P-values for these functional clusters were then false-discovery rate adjusted (FDR) to correct for multiple testing, with the FDR adjusted p-value of 0.05 considered the threshold for significance.

For the eQTL analysis, 501 participants with complete genotyping data from the InChianti study were also successfully assayed on Illumina HT12v.3 genome-wide expression arrays using RNA isolated from whole blood. Quality control of the genome-wide expression data included the exclusion of probes with detection p-values>0.01 with missing data for greater than 5% of participants. Samples must have been assayed with at least 95% of the filtered probe sets passing quality control in order to be included in analyses.

5094 probes passed quality control and were subsequently cubic-spline normalized prior to analysis. In the investigation of possible cis-eQTL associations at regions of interest identified in the meta-analysis, all probes within 500 kb of successfully replicated SNPs from the meta-analysis were identified based on annotations from ReMOAT (http://www.compbio.group.cam.ac.uk/Resources/Annotation/) [83]. Thus, we tested 741 possible cis-eQTLs. Multivariate linear regression was implemented using PLINKv.1.07 [84], testing the dosage of minor alleles as a predictor of gene expression level for each probe. These linear regression models were adjusted for hybridization batch, amplification batches, sex, smoking, study site and age at baseline of study. The p-values generated by each analysis was corrected for the number of tests, with a minimum threshold of significance at p-value≤6.75E-05.

## Supporting Information

Figure S1 Detailed association plot for the WBC locus at Chr6:31033692–31233692 bp. Locus specific plots showing top SNP per replicated locus +/−100 kilobases. SNPs in each region are color-coded based on linkage disequilibrium (r^2^) estimates from the CEU subset from HapMap Phase II: purple indicates reference SNP from meta-analysis, red indicates r^2^>0.8, orange indicates 0.6<r^2^≤0.8, green indicates 0.4<r^2^≤0.6, light blue indicates 0.2<r^2^≤0.4, and dark blue indicates r^2^≤0.2. Recombination rates estimated from the CEU HapMap Phase II data are included as a blue line in the background of the figure. Gene boundaries and exon positions are taken from RefSeq and UCSC Genome browser (build 36). Locus plots were generated using the LocusZoom Stand-alone package (http://genome.sph.umich.edu/wiki/LocusZoom_Standalone), incorporating the R packages Grid and Lattice, as well as the package New Fugue (http://genome.sph.umich.edu/wiki/New_Fugue) to estimate LD structure.
(PDF)

Figure S2 Detailed association plot for the WBC locus at Chr17:35310238–35510238 bp. Locus specific plots showing top SNP per replicated locus +/−100 kilobases. SNPs in each region are color-coded based on linkage disequilibrium (r^2^) estimates from the CEU subset from HapMap Phase II: purple indicates reference SNP from meta-analysis, red indicates r^2^>0.8, orange indicates 0.6<r^2^≤0.8, green indicates 0.4<r^2^≤0.6, light blue indicates 0.2<r^2^≤0.4, and dark blue indicates r^2^≤0.2. Recombination rates estimated from the CEU HapMap Phase II data are included as a blue line in the background of the figure. Gene boundaries and exon positions are taken from RefSeq and UCSC Genome browser (build 36). Locus plots were generated using the LocusZoom Stand-alone package (http://genome.sph.umich.edu/wiki/LocusZoom_Standalone), incorporating the R packages Grid and Lattice, as well as the package New Fugue (http://genome.sph.umich.edu/wiki/New_Fugue) to estimate LD structure.
(PDF)

Figure S3 Detailed association plot for the Neutrophil locus at Chr17:35306999–35506999 bp. Locus specific plots showing top SNP per replicated locus +/−100 kilobases. SNPs in each region are color-coded based on linkage disequilibrium (r^2^) estimates from the CEU subset from HapMap Phase II: purple indicates reference SNP from meta-analysis, red indicates r^2^>0.8, orange indicates 0.6<r^2^≤0.8, green indicates 0.4<r^2^≤0.6, light blue indicates 0.2<r^2^≤0.4, and dark blue indicates r^2^≤0.2. Recombination rates estimated from the CEU HapMap Phase II data are included as a blue line in the background of the figure. Gene boundaries and exon positions are taken from RefSeq and UCSC Genome browser (build 36). Locus plots were generated using the LocusZoom Stand-alone package (http://genome.sph.umich.edu/wiki/LocusZoom_Standalone), incorporating the R packages Grid and Lattice, as well as the package New Fugue (http://genome.sph.umich.edu/wiki/New_Fugue) to estimate LD structure.
(PDF)

Figure S4 Detailed association plot for the Basophil locus at Chr3:129699125–129899125 bp. Locus specific plots showing top SNP per replicated locus +/−100 kilobases. SNPs in each region are color-coded based on linkage disequilibrium (r^2^) estimates from the CEU subset from HapMap Phase II: purple indicates reference SNP from meta-analysis, red indicates r^2^>0.8, orange indicates 0.6<r^2^≤0.8, green indicates 0.4<r^2^≤0.6, light blue indicates 0.2<r^2^≤0.4, and dark blue indicates r^2^≤0.2. Recombination rates estimated from the CEU HapMap Phase II data are included as a blue line in the background of the figure. Gene boundaries and exon positions are taken from RefSeq and UCSC Genome browser (build 36). Locus plots were generated using the LocusZoom Stand-alone package (http://genome.sph.umich.edu/wiki/LocusZoom_Standalone), incorporating the R packages Grid and Lattice, as well as the package New Fugue (http://genome.sph.umich.edu/wiki/New_Fugue) to estimate LD structure.
(PDF)

Figure S5 Detailed association plot for the Lymphocyte locus at Chr6:31250153–31450153 bp. Locus specific plots showing top SNP per replicated locus +/−100 kilobases. SNPs in each region are color-coded based on linkage disequilibrium (r^2^) estimates from the CEU subset from HapMap Phase II: purple indicates reference SNP from meta-analysis, red indicates r^2^>0.8, orange indicates 0.6<r^2^≤0.8, green indicates 0.4<r^2^≤0.6, light blue indicates 0.2<r^2^≤0.4, and dark blue indicates r^2^≤0.2. Recombination rates estimated from the CEU HapMap Phase II data are included as a blue line in the background of the figure. Gene boundaries and exon positions are taken from RefSeq and UCSC Genome browser (build 36). Locus plots were generated using the LocusZoom Stand-alone package (http://genome.sph.umich.edu/wiki/LocusZoom_Standalone), incorporating the R packages Grid and Lattice, as well as the package New Fugue (http://genome.sph.umich.edu/wiki/New_Fugue) to estimate LD structure.
(PDF)

Figure S6 Detailed association plot for the Lymphocyte locus at Chr19:16309375–16509375 bp. Locus specific plots showing top SNP per replicated locus +/−100 kilobases. SNPs in each region are color-coded based on linkage disequilibrium (r^2^) estimates from the CEU subset from HapMap Phase II: purple indicates reference SNP from meta-analysis, red indicates r^2^>0.8, orange indicates 0.6<r^2^≤0.8, green indicates 0.4<r^2^≤0.6, light blue indicates 0.2<r^2^≤0.4, and dark blue indicates r^2^≤0.2. Recombination rates estimated from the CEU HapMap Phase II data are included as a blue line in the background of the figure. Gene boundaries and exon positions are taken from RefSeq and UCSC Genome browser (build 36). Locus plots were generated using the LocusZoom Stand-alone package (http://genome.sph.umich.edu/wiki/LocusZoom_Standalone), incorporating the R packages Grid and Lattice, as well as the package New Fugue (http://genome.sph.umich.edu/wiki/New_Fugue) to estimate LD structure.
(PDF)

Figure S7 Detailed association plot for the Monocyte locus at Chr2:181927546–182127546 bp. Locus specific plots showing top SNP per replicated locus +/−100 kilobases. SNPs in each region are color-coded based on linkage disequilibrium (r^2^) estimates from the CEU subset from HapMap Phase II: purple indicates reference SNP from meta-analysis, red indicates r^2^>0.8, orange indicates 0.6<r^2^≤0.8, green indicates 0.4<r^2^≤0.6, light blue indicates 0.2<r^2^≤0.4, and dark blue indicates r^2^≤0.2. Recombination rates estimated from the CEU HapMap Phase II data are included as a blue line in the background of the figure. Gene boundaries and exon positions are taken from RefSeq and UCSC Genome browser (build 36). Locus plots were generated using the LocusZoom Stand-alone package (http://genome.sph.umich.edu/wiki/LocusZoom_Standalone), incorporating the R packages Grid and Lattice, as well as the package New Fugue (http://genome.sph.umich.edu/wiki/New_Fugue) to estimate LD structure.
(PDF)

Figure S8 Detailed association plot for the Monocyte locus at Chr3:129680259–129880259 bp. Locus specific plots showing top SNP per replicated locus +/−100 kilobases. SNPs in each region are color-coded based on linkage disequilibrium (r^2^) estimates from the CEU subset from HapMap Phase II: purple indicates reference SNP from meta-analysis, red indicates r^2^>0.8, orange indicates 0.6<r^2^≤0.8, green indicates 0.4<r^2^≤0.6, light blue indicates 0.2<r^2^≤0.4, and dark blue indicates r^2^≤0.2. Recombination rates estimated from the CEU HapMap Phase II data are included as a blue line in the background of the figure. Gene boundaries and exon positions are taken from RefSeq and UCSC Genome browser (build 36). Locus plots were generated using the LocusZoom Stand-alone package (http://genome.sph.umich.edu/wiki/LocusZoom_Standalone), incorporating the R packages Grid and Lattice, as well as the package New Fugue (http://genome.sph.umich.edu/wiki/New_Fugue) to estimate LD structure.
(PDF)

Figure S9 Detailed association plot for the Monocyte locus at Chr8:130578550–130778550 bp. Locus specific plots showing top SNP per replicated locus +/−100 kilobases. SNPs in each region are color-coded based on linkage disequilibrium (r^2^) estimates from the CEU subset from HapMap Phase II: purple indicates reference SNP from meta-analysis, red indicates r^2^>0.8, orange indicates 0.6<r^2^≤0.8, green indicates 0.4<r^2^≤0.6, light blue indicates 0.2<r^2^≤0.4, and dark blue indicates r^2^≤0.2. Recombination rates estimated from the CEU HapMap Phase II data are included as a blue line in the background of the figure. Gene boundaries and exon positions are taken from RefSeq and UCSC Genome browser (build 36). Locus plots were generated using the LocusZoom Stand-alone package (http://genome.sph.umich.edu/wiki/LocusZoom_Standalone), incorporating the R packages Grid and Lattice, as well as the package New Fugue (http://genome.sph.umich.edu/wiki/New_Fugue) to estimate LD structure.
(PDF)

Figure S10 Detailed association plot for the Monocyte locus at Chr9:112855726–113055726 bp. Locus specific plots showing top SNP per replicated locus +/−100 kilobases. SNPs in each region are color-coded based on linkage disequilibrium (r^2^) estimates from the CEU subset from HapMap Phase II: purple indicates reference SNP from meta-analysis, red indicates r^2^>0.8, orange indicates 0.6<r^2^≤0.8, green indicates 0.4<r^2^≤0.6, light blue indicates 0.2<r^2^≤0.4, and dark blue indicates r^2^≤0.2. Recombination rates estimated from the CEU HapMap Phase II data are included as a blue line in the background of the figure. Gene boundaries and exon positions are taken from RefSeq and UCSC Genome browser (build 36). Locus plots were generated using the LocusZoom Stand-alone package (http://genome.sph.umich.edu/wiki/LocusZoom_Standalone), incorporating the R packages Grid and Lattice, as well as the package New Fugue (http://genome.sph.umich.edu/wiki/New_Fugue) to estimate LD structure.
(PDF)

Table S1 Genomic inflation factors λ_GC_ for discovery stage analyses. The λ_GC_ values were calculated inclusive of all SNPs analyzed, and these values were utilized as genomic control factors for the meta-analyses.
(PDF)

Table S2 Full discovery findings and replication results for SNPs of interest from the discovery stage of analyses.
(XLS)

Table S3 Results of fixed-effects meta-analyses incorperating a study-level adjustment for the most significant SNP per locus identified.
(TXT)

Table S4 Results of fixed-effects meta-analyses incorperating a study-level adjustment for the total WBC measure.
(TXT)

Table S5 Adjusted r^2^ estimates across phenotypes.
(XLSX)

Table S6 Gene based clustering from GRAIL. This includes all clusters evaluated.
(PDF)

Table S7 eQTL analysis results for all associations tested between SNPs and transcripts in a subset of 501 samples from the InCianti study.
(TXT)

Table S8 iHS scores per SNP of interest.
(PDF)

Text S1 Study descriptions and additional information.
(DOC)
