# Supplementary figures and images for: Multiple Loci Are Associated with White Blood Cell Phenotypes
Source: PLoS Genet. 2011 Jun 30;7(6):e1002113. doi: 10.1371/journal.pgen.1002113 (PMC3128114; doi:10.1371/journal.pgen.1002113)

# WBC Chr6:31033692-31233692

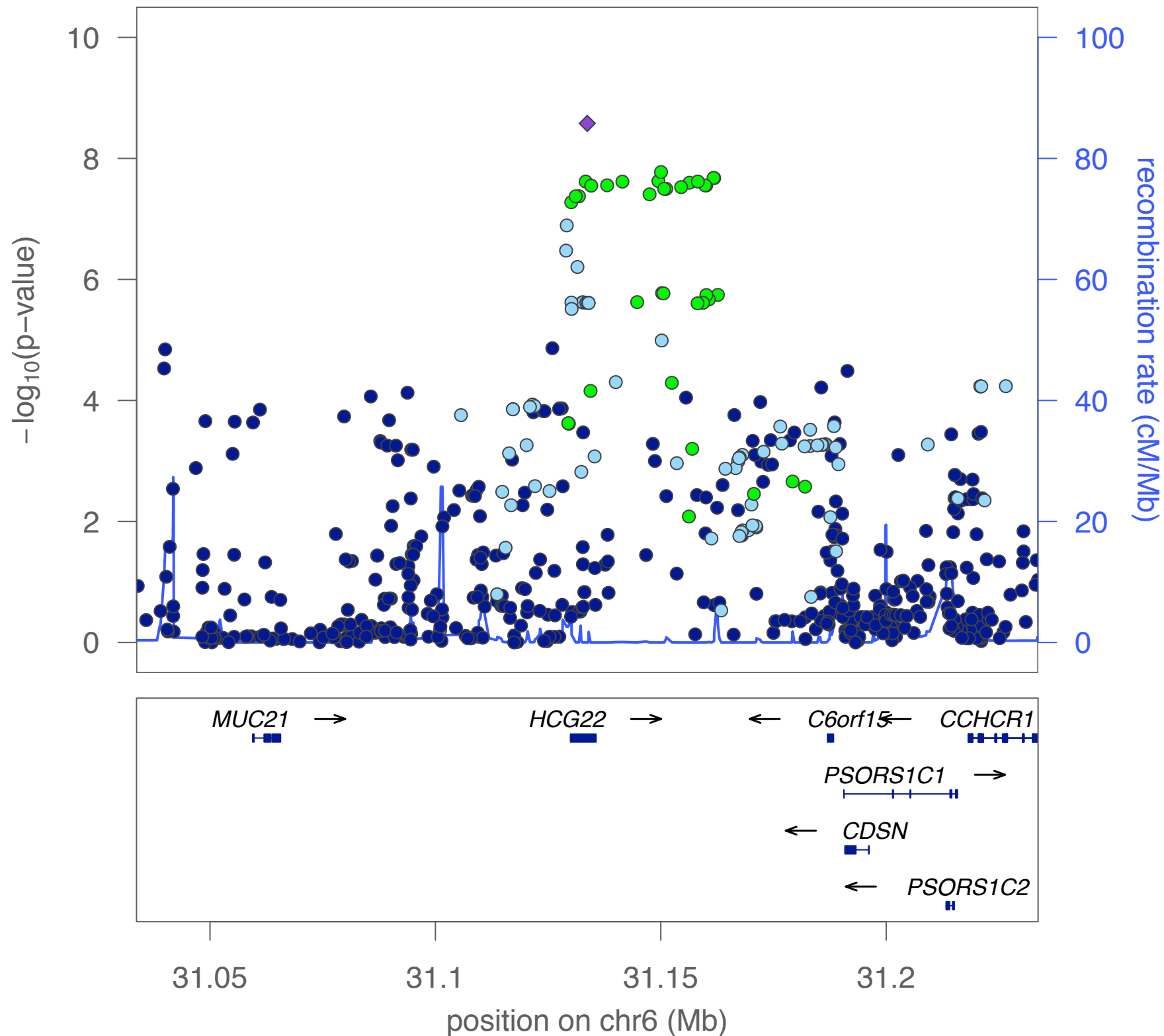

Supplement: Figure S1 — Detailed association plot for the WBC locus at Chr6:31033692–31233692 bp. Locus specific plots showing top SNP per replicated locus +/−100 kilobases. SNPs in each region are color-coded based on linkage disequilibrium (r2) estimates from the CEU subset from HapMap Phase II: purple indicates reference SNP from meta-analysis, red indicates r2>0.8, orange indicates 0.6<r2≤0.8, green indicates 0.4<r2≤0.6, light blue indicates 0.2<r2≤0.4, and dark blue indicates r2≤0.2. Recombination rates estimated from the CEU HapMap Phase II data are included as a blue line in the background of the figure. Gene boundaries and exon positions are taken from RefSeq and UCSC Genome browser (build 36). Locus plots were generated using the LocusZoom Stand-alone package (http://genome.sph.umich.edu/wiki/LocusZoom_Standalone), incorporating the R packages Grid and Lattice, as well as the package New Fugue (http://genome.sph.umich.edu/wiki/New_Fugue) to estimate LD structure. (PDF) [file pgen.1002113.s001.pdf]

# WBC Chr17:35310238–35510238

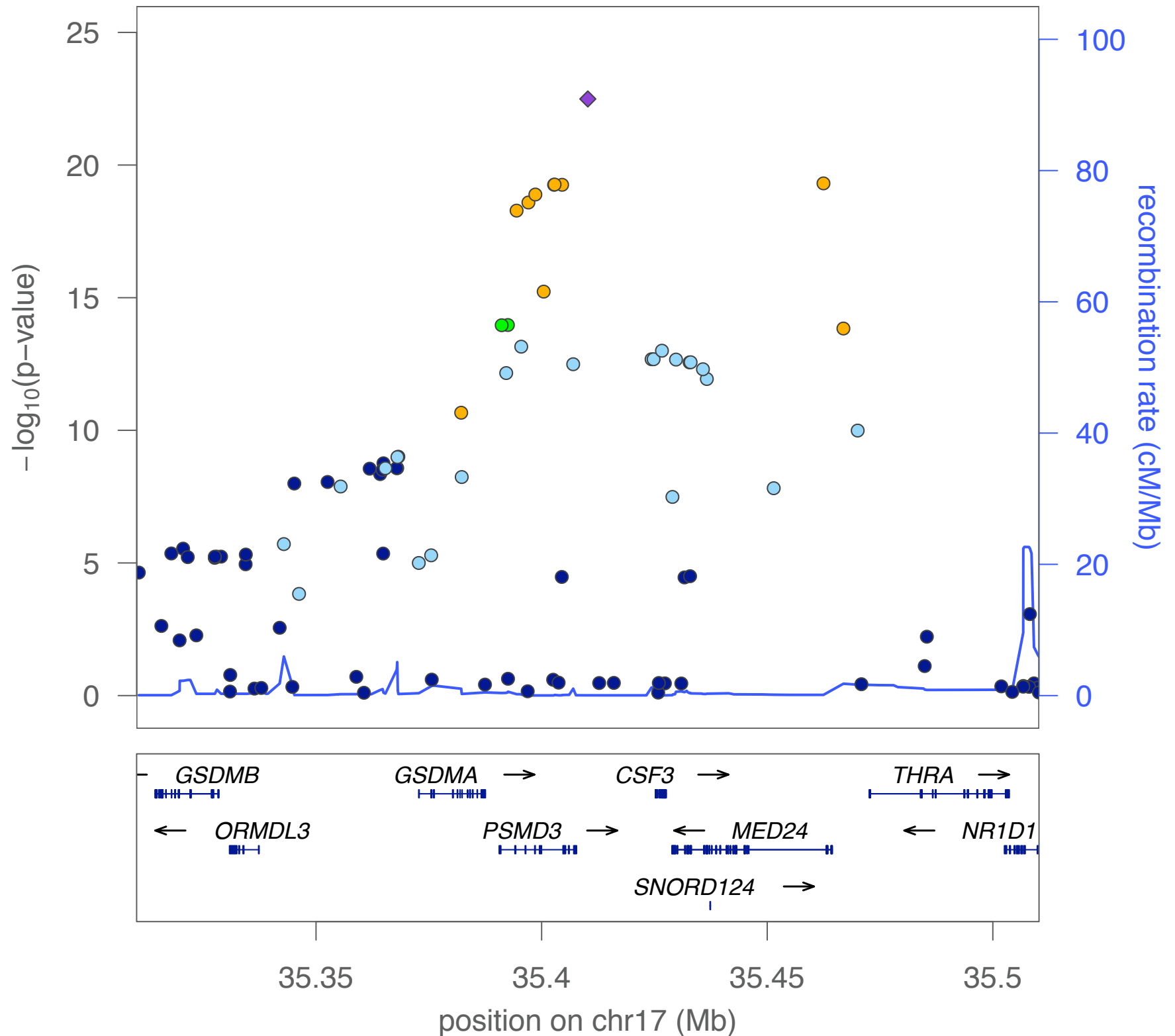

Supplement: Figure S2 — Detailed association plot for the WBC locus at Chr17:35310238–35510238 bp. Locus specific plots showing top SNP per replicated locus +/−100 kilobases. SNPs in each region are color-coded based on linkage disequilibrium (r2) estimates from the CEU subset from HapMap Phase II: purple indicates reference SNP from meta-analysis, red indicates r2>0.8, orange indicates 0.6<r2≤0.8, green indicates 0.4<r2≤0.6, light blue indicates 0.2<r2≤0.4, and dark blue indicates r2≤0.2. Recombination rates estimated from the CEU HapMap Phase II data are included as a blue line in the background of the figure. Gene boundaries and exon positions are taken from RefSeq and UCSC Genome browser (build 36). Locus plots were generated using the LocusZoom Stand-alone package (http://genome.sph.umich.edu/wiki/LocusZoom_Standalone), incorporating the R packages Grid and Lattice, as well as the package New Fugue (http://genome.sph.umich.edu/wiki/New_Fugue) to estimate LD structure. (PDF) [file pgen.1002113.s002.pdf]

# Neutrophils Chr17:35306999–35506999

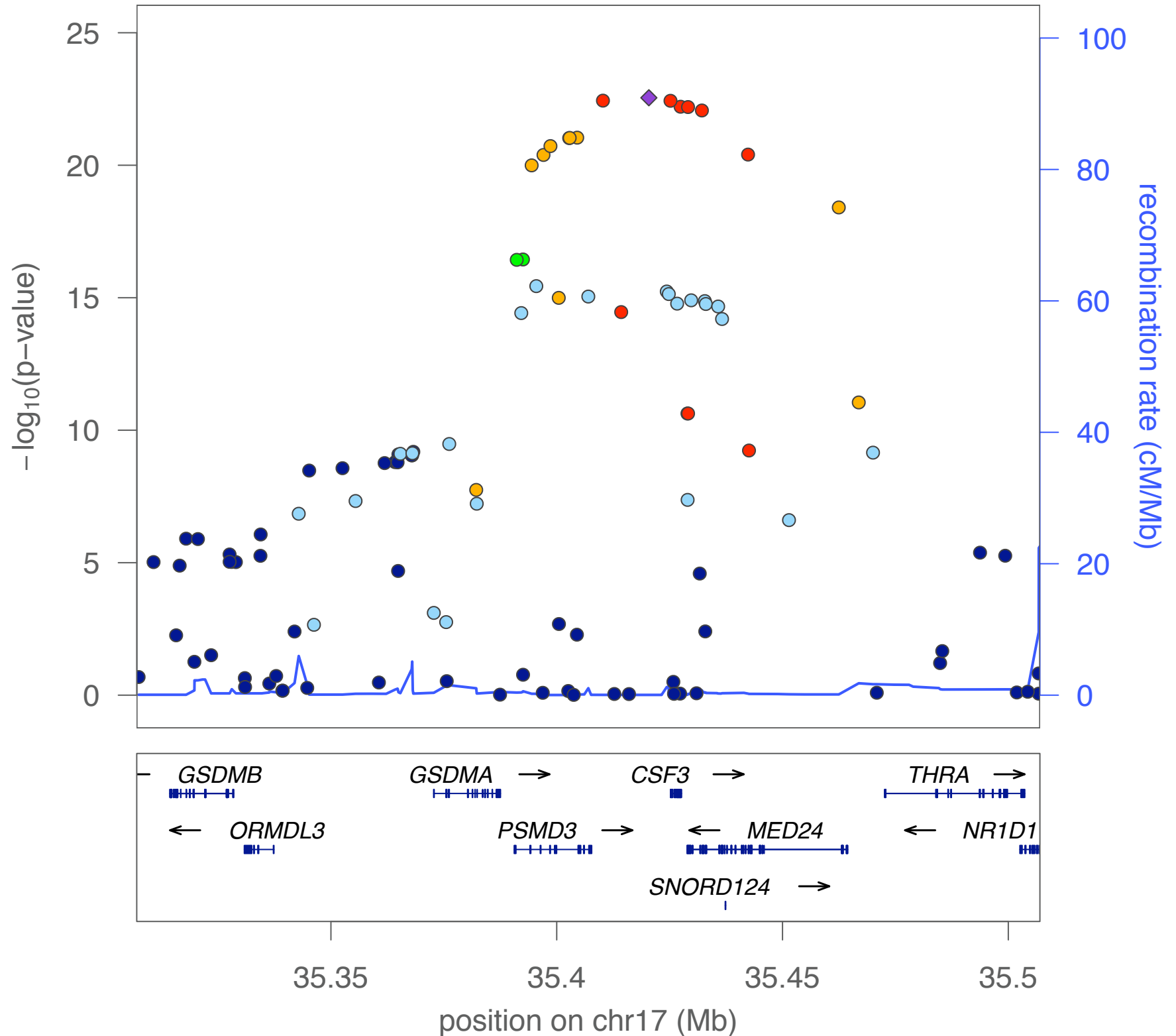

Supplement: Figure S3 — Detailed association plot for the Neutrophil locus at Chr17:35306999–35506999 bp. Locus specific plots showing top SNP per replicated locus +/−100 kilobases. SNPs in each region are color-coded based on linkage disequilibrium (r2) estimates from the CEU subset from HapMap Phase II: purple indicates reference SNP from meta-analysis, red indicates r2>0.8, orange indicates 0.6<r2≤0.8, green indicates 0.4<r2≤0.6, light blue indicates 0.2<r2≤0.4, and dark blue indicates r2≤0.2. Recombination rates estimated from the CEU HapMap Phase II data are included as a blue line in the background of the figure. Gene boundaries and exon positions are taken from RefSeq and UCSC Genome browser (build 36). Locus plots were generated using the LocusZoom Stand-alone package (http://genome.sph.umich.edu/wiki/LocusZoom_Standalone), incorporating the R packages Grid and Lattice, as well as the package New Fugue (http://genome.sph.umich.edu/wiki/New_Fugue) to estimate LD structure. (PDF) [file pgen.1002113.s003.pdf]

# Basophils Chr3:129699125–129899125

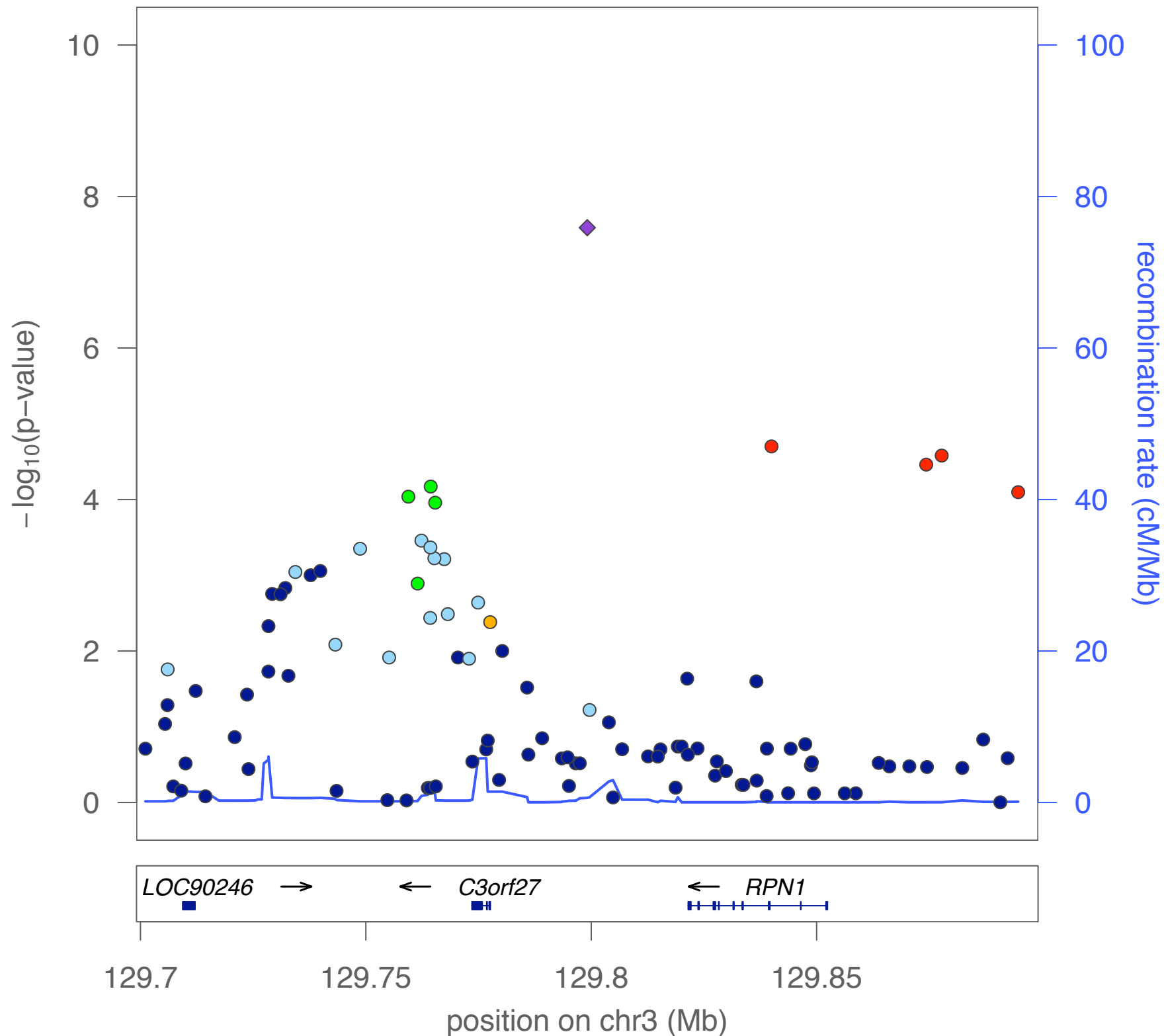

Supplement: Figure S4 — Detailed association plot for the Basophil locus at Chr3:129699125–129899125 bp. Locus specific plots showing top SNP per replicated locus +/−100 kilobases. SNPs in each region are color-coded based on linkage disequilibrium (r2) estimates from the CEU subset from HapMap Phase II: purple indicates reference SNP from meta-analysis, red indicates r2>0.8, orange indicates 0.6<r2≤0.8, green indicates 0.4<r2≤0.6, light blue indicates 0.2<r2≤0.4, and dark blue indicates r2≤0.2. Recombination rates estimated from the CEU HapMap Phase II data are included as a blue line in the background of the figure. Gene boundaries and exon positions are taken from RefSeq and UCSC Genome browser (build 36). Locus plots were generated using the LocusZoom Stand-alone package (http://genome.sph.umich.edu/wiki/LocusZoom_Standalone), incorporating the R packages Grid and Lattice, as well as the package New Fugue (http://genome.sph.umich.edu/wiki/New_Fugue) to estimate LD structure. (PDF) [file pgen.1002113.s004.pdf]

# Lymphocytes Chr6:31250153–31450153

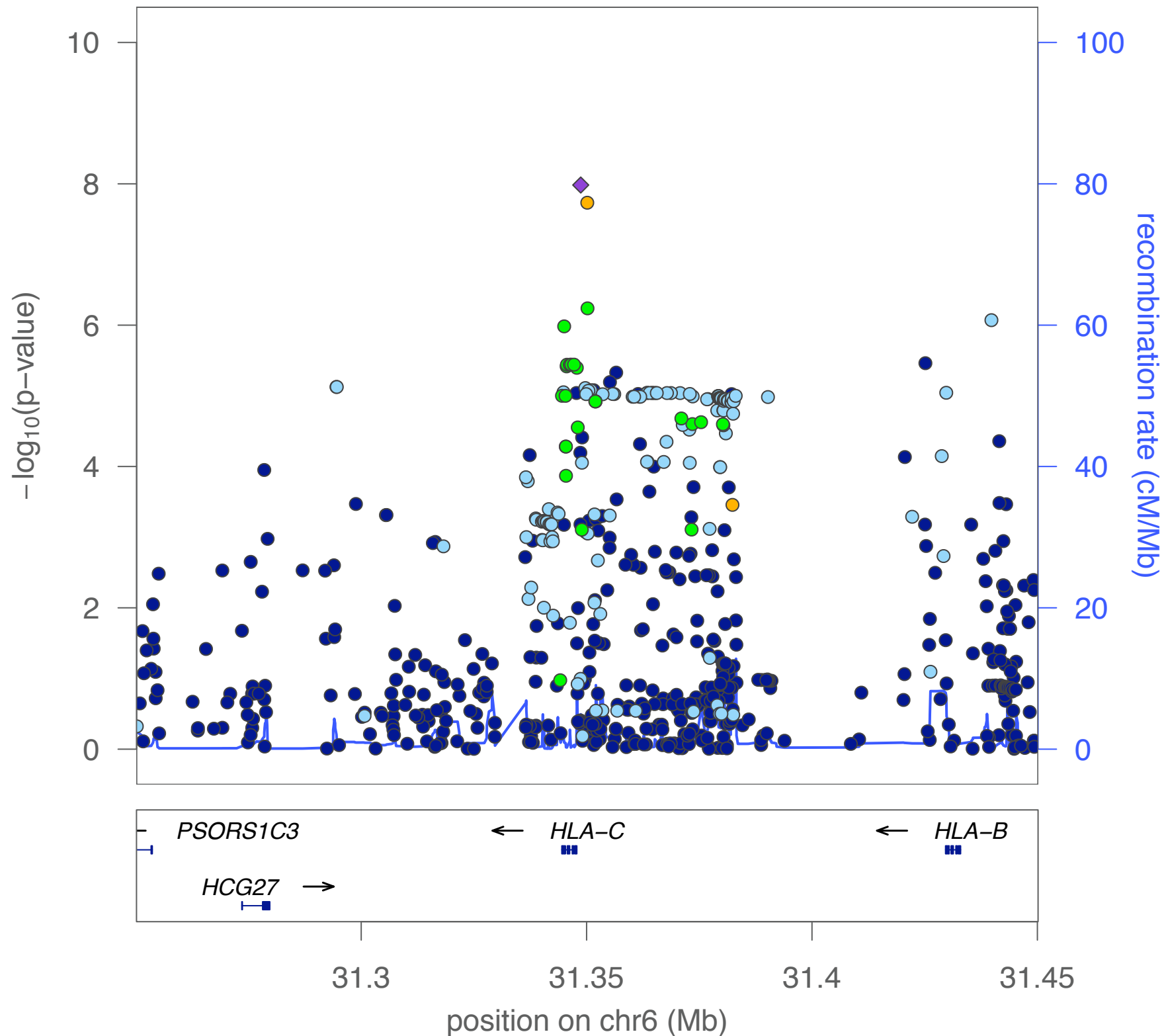

Supplement: Figure S5 — Detailed association plot for the Lymphocyte locus at Chr6:31250153–31450153 bp. Locus specific plots showing top SNP per replicated locus +/−100 kilobases. SNPs in each region are color-coded based on linkage disequilibrium (r2) estimates from the CEU subset from HapMap Phase II: purple indicates reference SNP from meta-analysis, red indicates r2>0.8, orange indicates 0.6<r2≤0.8, green indicates 0.4<r2≤0.6, light blue indicates 0.2<r2≤0.4, and dark blue indicates r2≤0.2. Recombination rates estimated from the CEU HapMap Phase II data are included as a blue line in the background of the figure. Gene boundaries and exon positions are taken from RefSeq and UCSC Genome browser (build 36). Locus plots were generated using the LocusZoom Stand-alone package (http://genome.sph.umich.edu/wiki/LocusZoom_Standalone), incorporating the R packages Grid and Lattice, as well as the package New Fugue (http://genome.sph.umich.edu/wiki/New_Fugue) to estimate LD structure. (PDF) [file pgen.1002113.s005.pdf]

# Lymphocytes Chr19:16309375–16509375

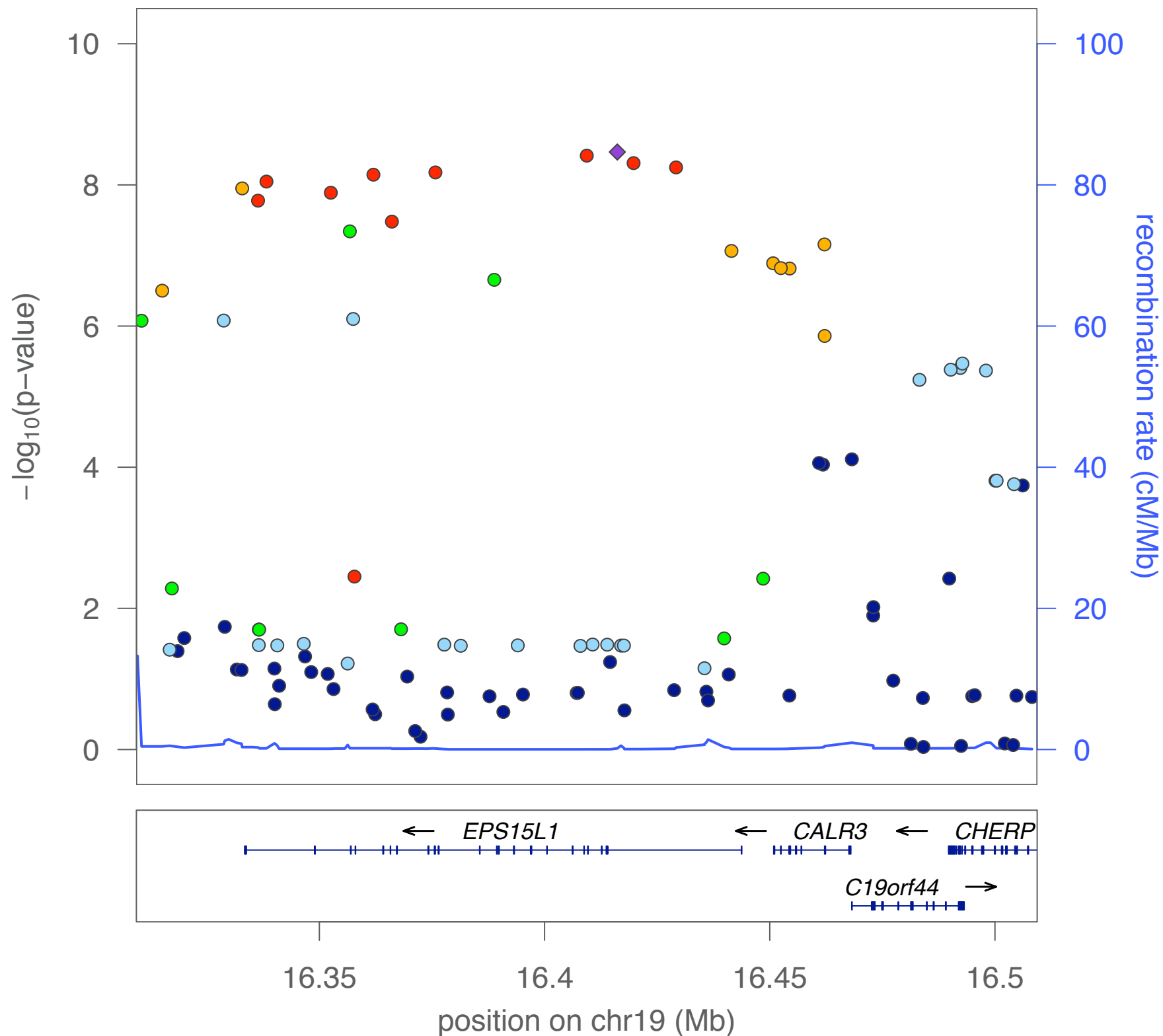

Supplement: Figure S6 — Detailed association plot for the Lymphocyte locus at Chr19:16309375–16509375 bp. Locus specific plots showing top SNP per replicated locus +/−100 kilobases. SNPs in each region are color-coded based on linkage disequilibrium (r2) estimates from the CEU subset from HapMap Phase II: purple indicates reference SNP from meta-analysis, red indicates r2>0.8, orange indicates 0.6<r2≤0.8, green indicates 0.4<r2≤0.6, light blue indicates 0.2<r2≤0.4, and dark blue indicates r2≤0.2. Recombination rates estimated from the CEU HapMap Phase II data are included as a blue line in the background of the figure. Gene boundaries and exon positions are taken from RefSeq and UCSC Genome browser (build 36). Locus plots were generated using the LocusZoom Stand-alone package (http://genome.sph.umich.edu/wiki/LocusZoom_Standalone), incorporating the R packages Grid and Lattice, as well as the package New Fugue (http://genome.sph.umich.edu/wiki/New_Fugue) to estimate LD structure. (PDF) [file pgen.1002113.s006.pdf]

# Monocytes Chr2:181927546–182127546

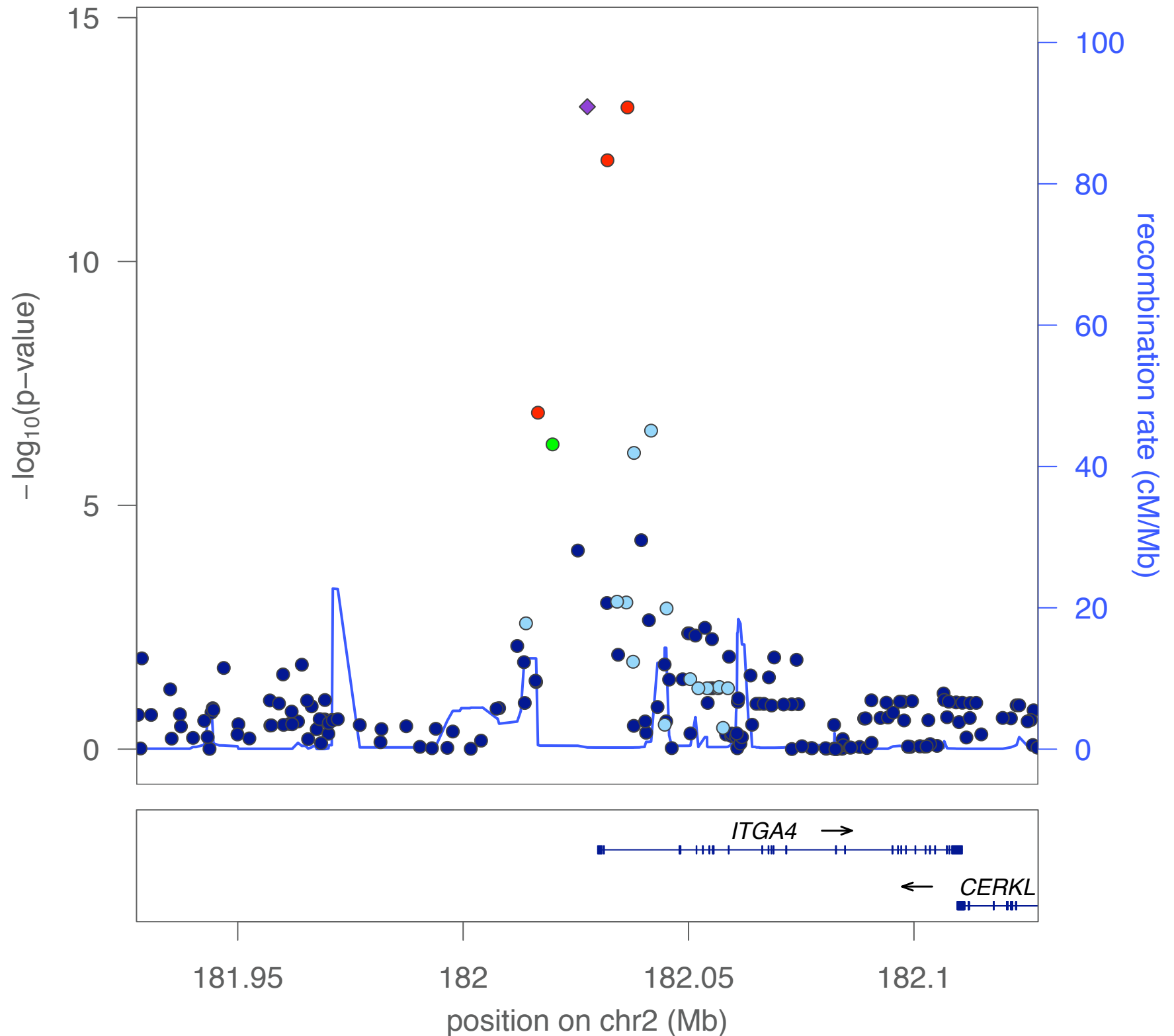

Supplement: Figure S7 — Detailed association plot for the Monocyte locus at Chr2:181927546–182127546 bp. Locus specific plots showing top SNP per replicated locus +/−100 kilobases. SNPs in each region are color-coded based on linkage disequilibrium (r2) estimates from the CEU subset from HapMap Phase II: purple indicates reference SNP from meta-analysis, red indicates r2>0.8, orange indicates 0.6<r2≤0.8, green indicates 0.4<r2≤0.6, light blue indicates 0.2<r2≤0.4, and dark blue indicates r2≤0.2. Recombination rates estimated from the CEU HapMap Phase II data are included as a blue line in the background of the figure. Gene boundaries and exon positions are taken from RefSeq and UCSC Genome browser (build 36). Locus plots were generated using the LocusZoom Stand-alone package (http://genome.sph.umich.edu/wiki/LocusZoom_Standalone), incorporating the R packages Grid and Lattice, as well as the package New Fugue (http://genome.sph.umich.edu/wiki/New_Fugue) to estimate LD structure. (PDF) [file pgen.1002113.s007.pdf]

# Monocytes Chr3:129680259–129880259

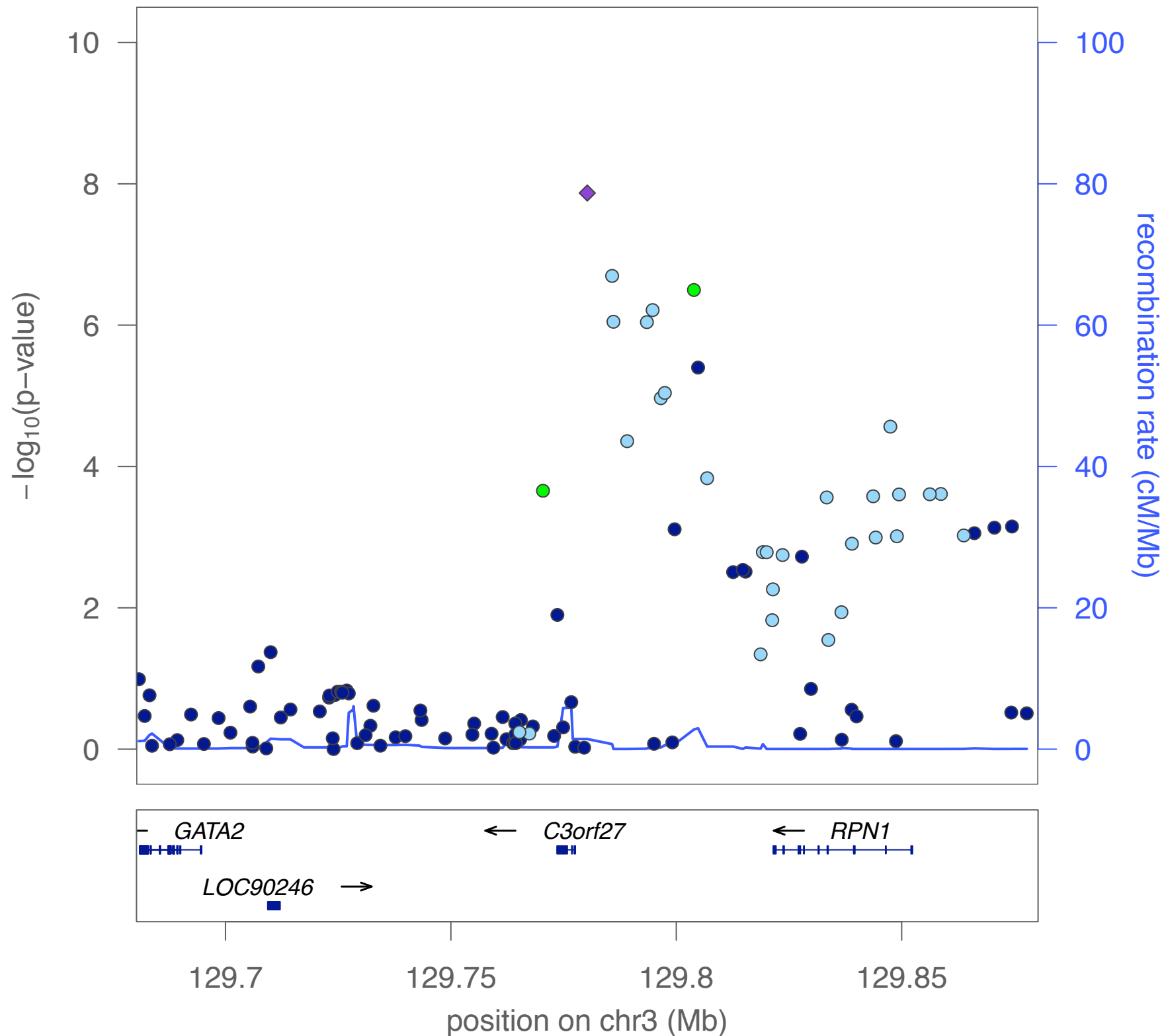

Supplement: Figure S8 — Detailed association plot for the Monocyte locus at Chr3:129680259–129880259 bp. Locus specific plots showing top SNP per replicated locus +/−100 kilobases. SNPs in each region are color-coded based on linkage disequilibrium (r2) estimates from the CEU subset from HapMap Phase II: purple indicates reference SNP from meta-analysis, red indicates r2>0.8, orange indicates 0.6<r2≤0.8, green indicates 0.4<r2≤0.6, light blue indicates 0.2<r2≤0.4, and dark blue indicates r2≤0.2. Recombination rates estimated from the CEU HapMap Phase II data are included as a blue line in the background of the figure. Gene boundaries and exon positions are taken from RefSeq and UCSC Genome browser (build 36). Locus plots were generated using the LocusZoom Stand-alone package (http://genome.sph.umich.edu/wiki/LocusZoom_Standalone), incorporating the R packages Grid and Lattice, as well as the package New Fugue (http://genome.sph.umich.edu/wiki/New_Fugue) to estimate LD structure. (PDF) [file pgen.1002113.s008.pdf]

# Monocytes Chr8:130578550–130778550

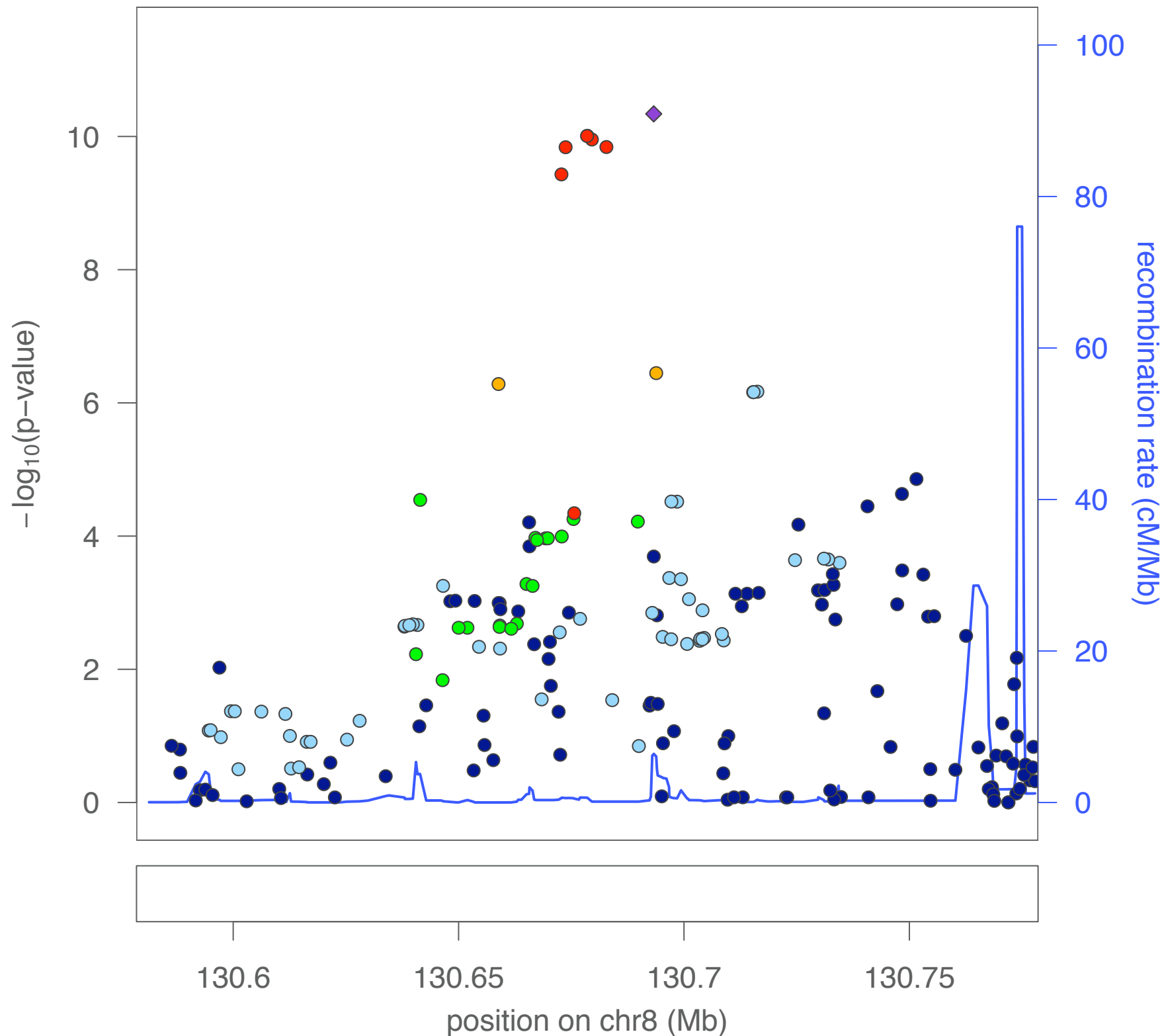

Supplement: Figure S9 — Detailed association plot for the Monocyte locus at Chr8:130578550–130778550 bp. Locus specific plots showing top SNP per replicated locus +/−100 kilobases. SNPs in each region are color-coded based on linkage disequilibrium (r2) estimates from the CEU subset from HapMap Phase II: purple indicates reference SNP from meta-analysis, red indicates r2>0.8, orange indicates 0.6<r2≤0.8, green indicates 0.4<r2≤0.6, light blue indicates 0.2<r2≤0.4, and dark blue indicates r2≤0.2. Recombination rates estimated from the CEU HapMap Phase II data are included as a blue line in the background of the figure. Gene boundaries and exon positions are taken from RefSeq and UCSC Genome browser (build 36). Locus plots were generated using the LocusZoom Stand-alone package (http://genome.sph.umich.edu/wiki/LocusZoom_Standalone), incorporating the R packages Grid and Lattice, as well as the package New Fugue (http://genome.sph.umich.edu/wiki/New_Fugue) to estimate LD structure. (PDF) [file pgen.1002113.s009.pdf]

# Monocytes Chr9:112855726–113055726

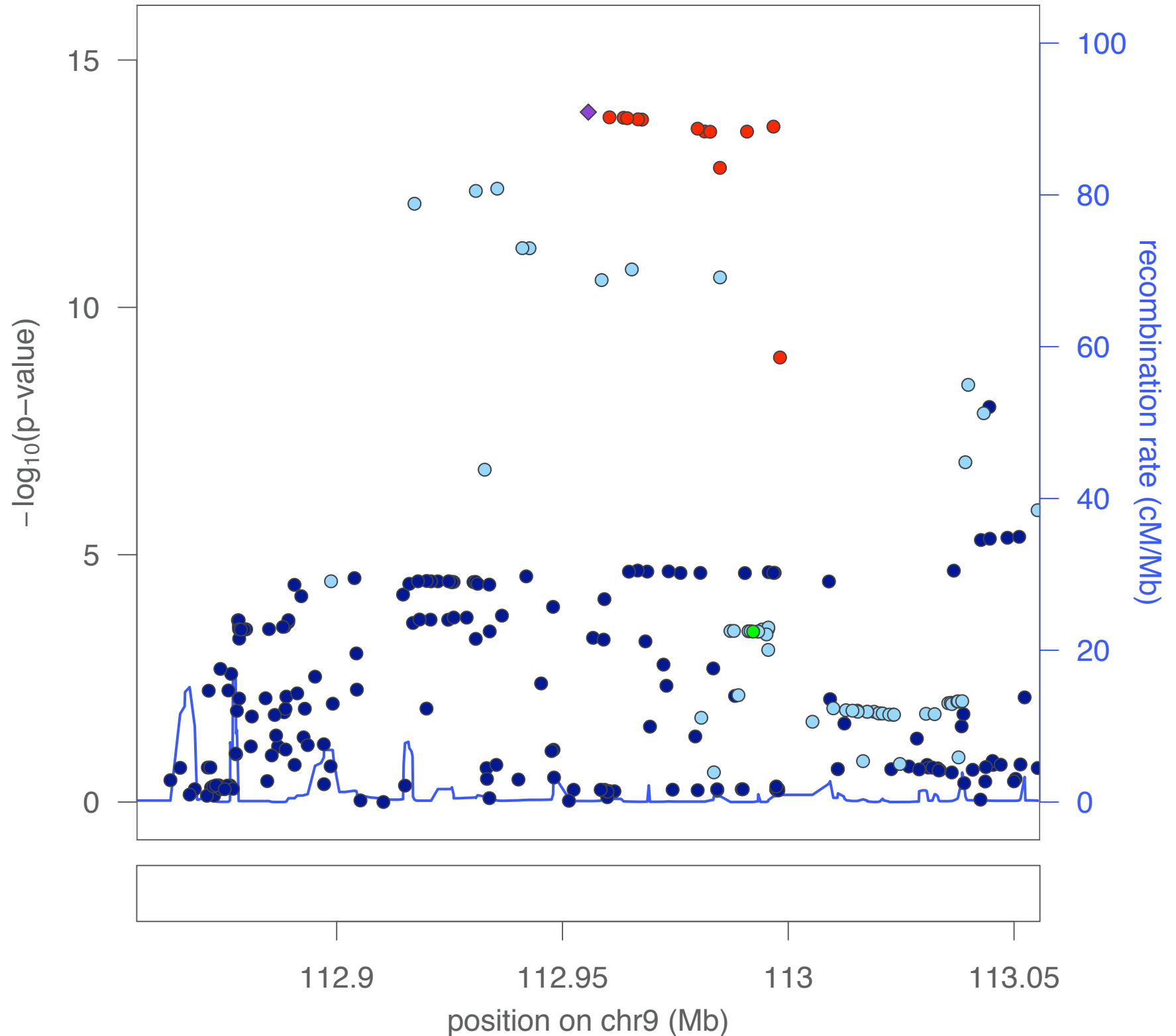

Supplement: Figure S10 — Detailed association plot for the Monocyte locus at Chr9:112855726–113055726 bp. Locus specific plots showing top SNP per replicated locus +/−100 kilobases. SNPs in each region are color-coded based on linkage disequilibrium (r2) estimates from the CEU subset from HapMap Phase II: purple indicates reference SNP from meta-analysis, red indicates r2>0.8, orange indicates 0.6<r2≤0.8, green indicates 0.4<r2≤0.6, light blue indicates 0.2<r2≤0.4, and dark blue indicates r2≤0.2. Recombination rates estimated from the CEU HapMap Phase II data are included as a blue line in the background of the figure. Gene boundaries and exon positions are taken from RefSeq and UCSC Genome browser (build 36). Locus plots were generated using the LocusZoom Stand-alone package (http://genome.sph.umich.edu/wiki/LocusZoom_Standalone), incorporating the R packages Grid and Lattice, as well as the package New Fugue (http://genome.sph.umich.edu/wiki/New_Fugue) to estimate LD structure. (PDF) [file pgen.1002113.s010.pdf]
