# Supplementary material for: Multiple Loci Are Associated with White Blood Cell Phenotypes
Source: PLoS Genet. 2011 Jun 30;7(6):e1002113. doi: 10.1371/journal.pgen.1002113 (PMC3128114; doi:10.1371/journal.pgen.1002113)
Supplement: Table S1 — Genomic inflation factors λGC for discovery stage analyses. The λGC values were calculated inclusive of all SNPs analyzed, and these values were utilized as genomic control factors for the meta-analyses. (PDF) [file pgen.1002113.s011.pdf]

Phenotype/Study	AGES	ARIC	BLSA	FHS	Health ABC	InChianti	RS	Meta-analysis
WBC	1.094	1.026	1.032	1.005	1.007	1.054	1.017	1.022
Granulocytes								
Basophils	1.046	1.008	0.977	NA	1.121	0.977	NA	1.000
Eosinophils	1.024	0.993	0.977	NA	0.977	0.977	NA	1.010
Neutrophils	1.05	1.031	1.006	NA	0.977	1.029	NA	1.015
Non-granulocytes								
Lymphocytes	1.051	1.005	1.016	NA	0.977	0.977	1.02	1.022
Monocytes	1.055	1.006	1.012	NA	0.99	1.004	NA	1.019
