# Supplementary material for: Multiple Loci Are Associated with White Blood Cell Phenotypes
Source: PLoS Genet. 2011 Jun 30;7(6):e1002113. doi: 10.1371/journal.pgen.1002113 (PMC3128114; doi:10.1371/journal.pgen.1002113)
Supplement: Table S6 — Gene based clustering from GRAIL. This includes all clusters evaluated. (PDF) [file pgen.1002113.s016.pdf]

Primary Gene	FDR adjusted P-value	Related genes from text mining with ranked keyword occurrence in parentheses
MLZE	0.002578089	GSDM1(1), GSDML(50), C19orf44(165), FBXL20(488), FAM49B(602)
GSDM1	0.003492814	C17orf37(2), GSDML(3), ZPBP2(4), GRB7(5), PERLD1(6), MLZE(9), ERBB2(24), FAM49B(431)
MED24	0.031794413	MED1(11), MED26(20), THRA(98), CRKRS(253)
MED1	0.041195074	MED24(3), MED26(21), THRA(38), CRKRS(311)
MED26	0.049341776	MED24(20), MED1(44), CRKRS(331), RPN1(404)
CALR3	0.053235178	HLA-C(86), HLA-B(88), FAM49B(230)
CSF3	0.058163179	ITGA4(196), HLA-C(526), ERBB2(614), POU5F1(621)
STARD3	0.07017308	RPN1(88), PSMD3(494), CRKRS(597)
TCF19	0.080379764	HLA-C(1), PSORS1C2(2), CDSN(13), HLA-B(18), C6orf15(28), CCHCR1(30), PSORS1C1(47), RPN1(163), FBXL20(424), CRKRS(614)
HLA-C	0.10101982	HLA-B(2), PSORS1C1(27), PSORS1C2(35), MICA(44), CDSN(62), CCHCR1(64), TCF19(68), C6orf15(134), HCG27(183), PSMD3(302), RPN1(405), HCG22(475)
EDG2	0.1137693	FAM49B(324), ITGA4(572)
PNMT	0.13258812	C17orf37(31), PERLD1(105), PSMD3(167), RPN1(219), HLA-C(337), GSDM1(512)
C6orf15	0.14106459	PSORS1C2(2), HCG22(3), CCHCR1(4), PSORS1C1(5), CDSN(6), HLA-C(8), TCF19(9), HLA-B(23), HCG27(34), RPN1(355), FBXL20(489), FAM49B(601)
CDSN	0.22267963	PSORS1C2(1), CCHCR1(2), PSORS1C1(4), HLA-C(5), C6orf15(8), TCF19(9), HLA-B(11), HCG27(69), HCG22(92), MICA(112), RPN1(385), PSMD3(489), ORMDL3(499), CRKRS(594)
PSMD3	0.22712849	FBXL20(310), CRKRS(397), RPN1(514)
GRB7	0.23022918	GSDM1(3), C17orf37(5), ERBB2(11), ZPBP2(25), PERLD1(41), GSDML(74), CRKRS(233), ITGA4(449)
STAC2	0.23555136	C19orf44(304)
HLA-B	0.23845161	HLA-C(2), MICA(23), PSORS1C1(41), PSORS1C2(45), CDSN(65), TCF19(71), CCHCR1(107), C6orf15(161), HCG27(174), HCG22(355), CALR3(383), PSMD3(448), ORMDL3(465)
IKZF3	0.2411984	POU5F1(263), HLA-C(475), CRKRS(573)
PSORS1C1	0.25264255	PSORS1C2(2), CCHCR1(3), HLA-C(4), CDSN(6), HLA-B(8), C6orf15(11), TCF19(26), HCG27(29), HCG22(54), MICA(55), ORMDL3(57), GSDML(163)
C17orf37	0.28737799	GSDM1(1), ERBB2(2), PERLD1(6), GRB7(12), GSDML(19), ZPBP2(41), CRKRS(159), FBXL20(239), FAM49B(444)
THRA	0.29333468	MED24(14), MED1(25), NR1D1(139), NEUROD2(489)
GSDML	0.29770183	ORMDL3(1), GSDM1(4), ZPBP2(5), C17orf37(18), PERLD1(85), ERBB2(242), HCG27(249), FAM49B(258), HLA-C(346), PSORS1C1(562), MLZE(622)
C19orf44	0.29848117	STAC2(478)
MICA	0.32331176	HLA-B(2), HLA-C(3), PSORS1C1(69), CDSN(95), PSORS1C2(105), TCF19(126), HCG27(155), CCHCR1(177), ORMDL3(321), ERBB2(375), HCG22(379), GSDML(527), C6orf15(561)
PSORS1C2	0.33695857	PSORS1C1(2), CCHCR1(3), CDSN(4), HLA-C(5), C6orf15(6), HLA-B(9), TCF19(10), HCG27(27), HCG22(40), MICA(60), ORMDL3(97), GSDML(202)
FAM49B	0.34954132	C19orf44(395)
POU5F1	0.36791912	NEUROD2(411)
HCG22	0.37134145	HCG27(4), C6orf15(6), PSORS1C1(35), HLA-C(42), PSORS1C2(46), CCHCR1(48), CDSN(51), HLA-B(86), ORMDL3(90), TCF19(472)
TCAP	0.38464911	PSMD3(534), RPN1(615)
CCHCR1	0.38713972	PSORS1C1(2), PSORS1C2(3), CDSN(4), HLA-C(5), C6orf15(6), TCF19(12), HLA-B(18), HCG22(29), HCG27(31), MICA(87), ORMDL3(129), GSDML(160), STARD3(338)
ERBB2	0.38991754	C17orf37(4), GRB7(180), GSDM1(197), CRKRS(446), HLA-C(582)
PERLD1	0.39804651	C17orf37(125), GSDM1(159), FBXL20(324)
NEUROD2	0.42549494	POU5F1(202)
ORMDL3	0.45004057	GSDML(2), FBXL20(147), HCG27(318)
SNORD124	1	SNORD124(7), FBXL20(555)
ZPBP2	1	GSDML(2), GSDM1(3), C17orf37(15), ORMDL3(18), GRB7(35), PERLD1(48), HCG27(361), STAC2(388)
ZNF483	1	
KIAA0368	1	FBXL20(362), CRKRS(572)
C3orf27	1	RPN1(5), GSDML(314), ERBB2(451), ORMDL3(463), HLA-C(555)
HCG27	1	HCG22(8), HLA-C(284), STAC2(299), PSORS1C1(313), ORMDL3(325), PSORS1C2(353), CDSN(498), HLA-B(503), C6orf15(530), CCHCR1(570)
OR2K2	1	FBXL20(547)
ITGA4	1	POU5F1(430), HLA-C(439)
CRKRS	1	FBXL20(141)
EPS15L1	1	CRKRS(155), FBXL20(278)
RPN1	1	FBXL20(264), CRKRS(437)
PPP1R1B	1	ERBB2(402), NEUROD2(531), CRKRS(540)
FBXL20	1	
NR1D1	1	THRA(22), MED24(64), MED1(65), CRKRS(557)
