# Supplementary material for: Multiple Loci Are Associated with White Blood Cell Phenotypes
Source: PLoS Genet. 2011 Jun 30;7(6):e1002113. doi: 10.1371/journal.pgen.1002113 (PMC3128114; doi:10.1371/journal.pgen.1002113)
Supplement: Table S8 — iHS scores per SNP of interest. (PDF) [file pgen.1002113.s018.pdf]

SNP	Phenotype	Discovery P-value	CHR	BP	iHS	Selection Type	Direction of Selection
rs4328821	Basophils	2.58E-08	3	129799125	-0.159181497	none	none
rs12536825	Eosinophils	7.97E-08	7	20470388	0.333762565	none	none
rs2524079	Lymphocytes	1.85E-08	6	31350153	-1.296173102	none	none
rs10402422	Lymphocytes	1.67E-08	19	16336388	-1.834123325	recent_moderate	negative
rs10404046	Lymphocytes	8.97E-09	19	16338255	-1.910718265	recent_moderate	negative
rs2034892	Lymphocytes	1.29E-08	19	16352549	-2.058966537	strong	negative
rs2290669	Lymphocytes	4.55E-08	19	16356774	-1.64110875	recent_moderate	negative
rs8110761	Lymphocytes	7.16E-09	19	16362005	-1.926778495	recent_moderate	negative
rs1870071	Lymphocytes	3.31E-08	19	16366106	-1.984842401	recent_moderate	negative
rs873636	Lymphocytes	6.65E-09	19	16375771	-2.139267684	strong	negative
rs10411936	Lymphocytes	3.85E-09	19	16409375	-1.91318907	recent_moderate	negative
rs11878602	Lymphocytes	3.42E-09	19	16416153	-1.92430769	recent_moderate	negative
rs7256818	Lymphocytes	4.91E-09	19	16419766	-1.824240107	recent_moderate	negative
rs2607278	Lymphocytes	5.65E-09	19	16429197	-1.803238268	recent_moderate	negative
rs1449263	Monocytes	6.71E-14	2	182027546	-0.326331027	none	none
rs1375493	Monocytes	8.40E-13	2	182032011	-0.431863965	none	none
rs2124440	Monocytes	6.93E-14	2	182036459	-0.734358567	none	none
rs9880192	Monocytes	1.35E-08	3	129780259	0.355951036	none	none
rs10107630	Monocytes	3.71E-10	8	130672817	-0.713146126	none	none
rs13277237	Monocytes	1.45E-10	8	130673745	-0.629216638	none	none
rs4480083	Monocytes	9.83E-11	8	130678502	-0.485323514	none	none
rs4407843	Monocytes	9.76E-11	8	130678550	-0.485323514	none	none
rs2163952	Monocytes	1.11E-10	8	130679571	1.475504671	none	none
rs10098310	Monocytes	1.44E-10	8	130682796	-0.381821443	none	none
rs1991866	Monocytes	4.58E-11	8	130693287	1.068696588	none	none
rs10817149	Monocytes	8.03E-13	9	112917232	0.488029973	none	none
rs1888190	Monocytes	4.43E-13	9	112930821	0.556419597	none	none
rs10980800	Monocytes	1.13E-14	9	112955726	-0.207062326	none	none
rs10980802	Monocytes	2.81E-11	9	112958677	-0.488977861	none	none
rs12346772	Monocytes	1.44E-14	9	112960420	-0.483739256	none	none
rs12350763	Monocytes	1.47E-14	9	112963544	-0.465755255	none	none
rs7034139	Monocytes	1.50E-14	9	112964338	-0.465755255	none	none
rs7023923	Monocytes	1.71E-11	9	112965355	1.043440839	none	none
rs1330279	Monocytes	1.58E-14	9	112966729	-0.479589102	none	none
rs12337595	Monocytes	1.60E-14	9	112967622	-0.479589102	none	none
rs10124626	Monocytes	2.44E-14	9	112979928	-0.48788941	none	none
rs10123393	Monocytes	2.78E-14	9	112981491	-0.450538024	none	none
rs17812386	Monocytes	2.82E-14	9	112982709	-0.450538024	none	none
rs12339649	Monocytes	1.51E-13	9	112984867	0.806975828	none	none
rs2150052	Monocytes	2.48E-11	9	112984888	1.174330765	none	none
rs17207270	Monocytes	2.80E-14	9	112990865	-0.472672179	none	none
rs12339151	Monocytes	2.22E-14	9	112996722	-0.280381712	none	none
rs2039183	Monocytes	1.03E-09	9	112998172	-0.223662942	none	none
rs824016	Monocytes	1.03E-08	9	113044530	-0.896224772	none	none
rs7207600	Neutrophils	3.33E-09	17	35345186	-0.080361431	none	none
rs8065126	Neutrophils	2.71E-09	17	35352561	-0.079033473	none	none
rs9895948	Neutrophils	1.74E-09	17	35361889	-0.196494185	none	none
rs17609240	Neutrophils	1.60E-09	17	35364215	1.050540958	none	none
rs8076474	Neutrophils	1.63E-09	17	35364760	-0.29399235	none	none
rs1007655	Neutrophils	8.18E-10	17	35364945	1.107439301	none	none
rs2313640	Neutrophils	7.76E-10	17	35365371	-0.223780878	none	none
rs7218742	Neutrophils	8.92E-10	17	35367887	-0.223780878	none	none

rs7218321	Neutrophils	8.83E-10	17	35367995	1.012245728	none	none
rs7219080	Neutrophils	7.42E-10	17	35368042	-0.222452921	none	none
rs6503527	Neutrophils	6.54E-10	17	35368245	-0.225108836	none	none
rs3859192	Neutrophils	1.79E-08	17	35382174	-0.089723143	none	none
rs8075668	Neutrophils	3.71E-17	17	35391149	0.631032855	none	none
rs2305481	Neutrophils	3.79E-15	17	35392150	-0.185489268	none	none
rs11655264	Neutrophils	3.60E-17	17	35392521	0.28715536	none	none
rs2305482	Neutrophils	1.01E-20	17	35394453	0.110907326	none	none
rs11078930	Neutrophils	3.65E-16	17	35395481	-0.782851133	none	none
rs4065321	Neutrophils	4.09E-21	17	35397074	0.79179698	none	none
rs9915252	Neutrophils	1.89E-21	17	35398614	0.177366169	none	none
rs8066582	Neutrophils	1.01E-15	17	35400455	0.167334646	none	none
rs11658328	Neutrophils	9.41E-22	17	35402762	0.445709421	none	none
rs8073254	Neutrophils	9.30E-22	17	35402876	0.445709421	none	none
rs2241245	Neutrophils	9.08E-22	17	35404540	0.331600842	none	none
rs12453334	Neutrophils	9.01E-16	17	35406999	-1.175926656	none	none
rs4794822	Neutrophils	3.64E-23	17	35410238	0.907439672	none	none
rs8070454	Neutrophils	3.48E-15	17	35414280	-0.010927767	none	none
rs8078723	Neutrophils	2.84E-23	17	35420405	-0.000656397	none	none
rs2227319	Neutrophils	5.82E-16	17	35424371	-1.143433811	none	none
rs2227321	Neutrophils	7.26E-16	17	35424820	-1.142116144	none	none
rs2227322	Neutrophils	3.69E-23	17	35425194	0.0173185	none	none
rs25645	Neutrophils	1.67E-15	17	35426669	-1.269929927	none	none
rs1042658	Neutrophils	6.18E-23	17	35427428	0.059687901	none	none
rs709592	Neutrophils	6.35E-23	17	35429079	0.059687901	none	none
rs2270401	Neutrophils	1.25E-15	17	35429782	-1.221176216	none	none
rs3213762	Neutrophils	8.56E-23	17	35432153	0.100773381	none	none
rs12451897	Neutrophils	1.34E-15	17	35432801	-1.044608721	none	none
rs2302777	Neutrophils	1.72E-15	17	35433018	1.512416013	none	none
rs9916158	Neutrophils	2.14E-15	17	35435755	-0.728368431	none	none
rs2302774	Neutrophils	6.34E-15	17	35436616	-0.594173516	none	none
rs7502514	Neutrophils	3.95E-21	17	35442370	-0.209632294	none	none
rs8065443	Neutrophils	3.91E-19	17	35462466	0.74660358	none	none
rs9262628	WBC	4.23E-08	6	31131127	-0.242585041	none	none
rs2523852	WBC	4.22E-08	6	31131847	-0.258410293	none	none
rs2517525	WBC	2.41E-08	6	31133340	-0.344840512	none	none
rs2517524	WBC	2.64E-09	6	31133692	-1.326108408	none	none
rs2523846	WBC	2.82E-08	6	31134568	-0.220673155	none	none
rs2517510	WBC	2.78E-08	6	31138101	-0.280322179	none	none
rs2517500	WBC	2.42E-08	6	31141488	1.137449276	none	none
rs2523890	WBC	3.92E-08	6	31147511	-0.171431457	none	none
rs2246330	WBC	2.37E-08	6	31149472	-0.486050447	none	none
rs2523883	WBC	1.68E-08	6	31150049	1.489266598	none	none
rs2523880	WBC	3.18E-08	6	31150748	-0.537178182	none	none
rs2249231	WBC	3.18E-08	6	31151211	-0.537178182	none	none
rs2523843	WBC	2.98E-08	6	31154507	-0.37405636	none	none
rs2249876	WBC	2.52E-08	6	31156366	-0.16954542	none	none
rs9263474	WBC	2.41E-08	6	31158223	1.258674721	none	none
rs2535317	WBC	2.79E-08	6	31159833	1.249451046	none	none
rs2517471	WBC	2.80E-08	6	31160077	-0.224325136	none	none
rs2535307	WBC	2.10E-08	6	31161720	-0.225542463	none	none
rs2535306	WBC	2.11E-08	6	31161846	1.204650338	none	none
rs2249742	WBC	1.73E-08	6	31348700	-1.156814348	none	none
rs7207600	WBC	1.01E-08	17	35345186	-0.080361431	none	none

rs8065126	WBC	8.84E-09	17	35352561	-0.079033473	none	none
rs4065985	WBC	1.31E-08	17	35355458	0.4023922	none	none
rs9895948	WBC	2.79E-09	17	35361889	-0.196494185	none	none
rs17609240	WBC	4.46E-09	17	35364215	1.050540958	none	none
rs8076474	WBC	3.12E-09	17	35364760	-0.29399235	none	none
rs1007655	WBC	1.75E-09	17	35364945	1.107439301	none	none
rs2313640	WBC	2.68E-09	17	35365371	-0.223780878	none	none
rs7218742	WBC	2.67E-09	17	35367887	-0.223780878	none	none
rs7218321	WBC	2.65E-09	17	35367995	1.012245728	none	none
rs7219080	WBC	1.02E-09	17	35368042	-0.222452921	none	none
rs6503527	WBC	9.88E-10	17	35368245	-0.225108836	none	none
rs3859192	WBC	2.17E-11	17	35382174	-0.089723143	none	none
rs8075668	WBC	1.09E-14	17	35391149	0.631032855	none	none
rs2305481	WBC	6.93E-13	17	35392150	-0.185489268	none	none
rs11655264	WBC	1.07E-14	17	35392521	0.28715536	none	none
rs2305482	WBC	5.22E-19	17	35394453	0.110907326	none	none
rs11078930	WBC	7.02E-14	17	35395481	-0.782851133	none	none
rs4065321	WBC	2.58E-19	17	35397074	0.79179698	none	none
rs9915252	WBC	1.29E-19	17	35398614	0.177366169	none	none
rs8066582	WBC	5.90E-16	17	35400455	0.167334646	none	none
rs11658328	WBC	5.53E-20	17	35402762	0.445709421	none	none
rs8073254	WBC	5.43E-20	17	35402876	0.445709421	none	none
rs2241245	WBC	5.55E-20	17	35404540	0.331600842	none	none
rs12453334	WBC	3.22E-13	17	35406999	-1.175926656	none	none
rs4794822	WBC	3.23E-23	17	35410238	0.907439672	none	none
rs2227319	WBC	2.09E-13	17	35424371	-1.143433811	none	none
rs2227321	WBC	2.09E-13	17	35424820	-1.142116144	none	none
rs25645	WBC	9.95E-14	17	35426669	-1.269929927	none	none
rs2270401	WBC	2.15E-13	17	35429782	-1.221176216	none	none
rs12451897	WBC	2.75E-13	17	35432801	-1.044608721	none	none
rs2302777	WBC	2.71E-13	17	35433018	1.512416013	none	none
rs9916158	WBC	4.97E-13	17	35435755	-0.728368431	none	none
rs2302774	WBC	1.16E-12	17	35436616	-0.594173516	none	none
rs11078936	WBC	1.52E-08	17	35451440	-0.374613523	none	none
rs8065443	WBC	4.91E-20	17	35462466	0.74660358	none	none
rs7502966	WBC	1.02E-10	17	35470048	0.072527114	none	none
