# Supplementary material for: Multiple Loci Are Associated with White Blood Cell Phenotypes
Source: PLoS Genet. 2011 Jun 30;7(6):e1002113. doi: 10.1371/journal.pgen.1002113 (PMC3128114; doi:10.1371/journal.pgen.1002113)
Supplement: Text S1 — Study descriptions and additional information. (DOC) [file pgen.1002113.s019.doc]

**STUDY INFORMATION**

This meta-analysis stems from collaborative efforts of the Cohorts for Heart and Aging Research in Genetic Epidemiology (CHARGE) consortium. Of the five principal cohorts of the CHARGE consortium, four studies possessed availible data for the analysis of white blood cell (WBC) phenotypes. We have additionally incorporated data from three additional studies based at the National Institute on Aging (NIA) at the National Institutes of Health. The seven combined CHARGE/NIA cohorts comprised the discovery meta-analysis. These seven studies conducted standardized study level analyses and planned replication of the initial results. Studies affiliated with the HeamGen and INGI groups agreed to contribute summary statistics as collaborators, permitting the replication of selected results from the discovery phase. CHARGE collaborators from the Heart and Vascular Health (HVH) and RSII also contributed additonal summary statistics to the replication efforts. All studies adhered to general phenotyping harmonization, exclusion criteria and analytic procedures described in the primary text of this manuscript. Any deviation from these methods is described in detail below. Each participating study was reviewed and approved by the corresponding IRB, and all subjects used for genetic analyses provided specific informed consent for genetic research. All participating studies approved guidelines for this collaboration, including data sharing and data security procedures. Individual study descriptions for both analytic stages follow below. All participating studies conducted their research in accordance with their respective institutional scientific and ethical review boards. All human participants provided informed consent and all clinical investigation was conducted in accordance with the Declaration of Helsinki.

*AGES*

The Reykjavik Study cohort originally comprised a random sample of 30,795 men and women born in 1907–1935 and living in Reykjavik in 1967. A total of 19381 attended, resulting in 71% recruitment rate. Between 2002 and 2006, the AGES-Reykjavik study re-examined 5764 survivors of the original Reykjavik Study cohort who had participated.

The AGES Reykjavik Study GWAS was approved by the National Bioethics Committee (00-063-V8+1) and the Data Protection Authority. DNA was genotyped using the Illumina 370CNV BeadChip array on 3,664 participants.  Samples were excluded from the dataset based on sample failure, genotype mismatch with reference panel, and sex mismatch, resulting in clean genotype data on 3,219 individuals. Standard protocols for working with Illumina data were followed, with clustering score greater than 0.4.   Prior to genotype imputation, SNPs were excluded using filters based on call rate (<97%), Hardy-Weinberg Equilibrium (p-value <1E-6), mishap (p-value <1E-9), and mismatched positions between Illumina, dbSNP and/or HapMap resulting in 325,094 SNPs passing all QC (of 353,202 prior to cleaning steps).  Imputation was done using MACH against all the HapMap CEPH haplotypes (release 22/NCBI build 36) resulting in 2,533,153 total SNPs for analysis.

WBC phenotypes were measured using fasting whole blood on a Beckman Coulter (HMX). Association analysis was conducted against all genotypes, and includes the most likely imputed gentoypes using ProbABEL.

*The Atherosclerosis Risk in Communities Study (ARIC)*

 The ARIC Study is a prospective population-based study of atherosclerotic disease in 15,792 mostly Caucasian and African American study participants sponsored by the National Heart, Lung, and Blood Institute (NHLBI). Participants aged 45-64 years were recruited by probability sampling and underwent a baseline examination and three subsequent examinations approximately every three years. Ancestry was self-reported, and only self-identified Caucasians were included in this analysis. A detailed study protocol is available on the ARIC study website (<http://www.cscc.unc.edu/aric>).

Data were collected from the baseline ARIC visit (1987-89). Blood was drawn following procedures described elsewhere. WBC and differential were measured using automated hematology analyzers: Coulter S+IV (calibration S-Cal, Beckman Coulter, Inc, Fullerton, CA) at 2 sites, Coulter S+III and Coulter S+IV (calibration S-Cal) at 1 site, and Technicon H-6000 (calibration Fisher, Technicon Corporation, Tarrytown, NY) at 1 site.

Genotypes were assayed using the Affymetrix Genome-Wide Human SNP Array 6.0 platform and genotypes were called using the Birdseed algorithm. For this analysis, the final sample with genotype data consisted of 8,861 samples from Caucasian participants who had provided informed consent for DNA studies. Genotype call rates were 95% or greater for all samples.

Of the 8,861 individuals, 734 were removed in data cleaning steps for sex mismatch, discordance with previously-genotyped markers, first-degree relative of an included individual, and genetic outlier based on allele sharing and principal components analyses. A further 5 individuals were removed because of missing values for current smoking status, leaving 8,122.  (There were no missing values for the other two covariates, age and gender.)  Next, 28 individuals were excluded for missing WBC data and 289 for WBC outside +/- 2 SD from the sample mean, leaving 7,805 individuals for analyses on WBC.  For WBC sub-type analysis, from the 8,122 individuals with cleaned and non-missing genotype and covariate data, 2,307 were excluded for missing values for any sub-type and a further 969 for a value outside +/- 2 SD from the sample mean for any sub-type, leaving 4,846 individuals for analyses on sub-types.  Data management and statistical analysis used SAS, R, ProbABEL and PLINK software.

*The Baltimore Longitudinal Study of Aging (BLSA)*

The BLSA is a community based longitudinal study of aging currently in its 51st year of follow-up. The study is comprised of volunteers recruited from an urban dwelling population based in Baltimore, MD. Overnight fasted blood samples were used for genomic extraction and hematological analyses at baseline. Hematological assays were carried out using a standard Coulter counter (Coulter Electronics, Hialeah, FL), with fasting blood sample aliquots stored at -80C and thawed  at time of assay. DNA extracted from BLSA participants was genotyped at the Laboratory of Neurogenetics, National Institute on Aging, Illumina Infinium technology (Illumina Inc., San Diego, CA). The assays used for genotyping included were the Infinium II HumanHap550 v. 1, Infinium II HumanHap550 v. 3, or a composite of Infinium HumanHap300 and Infinium II 240S. By combining the genotype data from Infinium HumanHap300 and Infinium II 240S assayed participants, to an equivalent level of genomic coverage as the Infinium II HumanHap550 assays, we were able to standardize participant data across a total of 545,066 single nucleotide polymorphisms genotyped on the Illumina platforms. After standard QC measures, genotypes were imputed using MACH (v.1.0.16) and linear regressiona nalyses were conducted using MACH2QTL.

*The Framingham Heart Study (FHS)*

The FHS is a prospective cohort study that aims to identify the risk factors for cardiovascular disease. Under the direction of the National Heart, Lung, and Blood Institute (NHLBI), the FHS recruited 5,209 men and women, the Original Cohort from the town of Framingham, Massachusetts in 1948; 5,124 of the second generation of the Original Cohort, the Offspring Cohort in 1971; and 4,095 participants of the Third Generation in 2002. The FHS cohort studied here is the Offspring Cohort, whose first exam was completed in 1975.

For FHS, the total WBC measure was the only phenotype availible for analyses and was measured at Exam 1 of the Offspring Cohort taken place between 1971 and 1975, as previously described. DNA was genotyped for the FHS participants with the Affymetrix 500K array and an additional gene-focused 50K array. MACH software was used to impute ~2.5 million SNPs based on the HapMap CEU phased haplotypes (build 22) and the SNPs that met the following criteria: minor allele frequency (MAF) > 0.01, Hardy Weinberg Equilibrium p-value >10E-6, SNP call rate > 97%, MISHAP test p-value >10E-9, Mendelian errors <= 100. Linear mixed effects model was used to account for familial correlation in the FHS GWAS, as FHS is the only study in this meta-analysis that included first degree relatives in its sample series.

*The Health, Aging and Body Composition Study (Health ABC)*

Health ABC is a cohort of 3075 community dwelling men and women recruited from known Medicare beneficiaries residing in the metropolitan areas of Pittsburgh, PA and Memphis, TN.  All particpants were selected to have high functional status at baseline.  Clinical and genetic analyses in Health ABC have been approved by institutional review boards of the University of Pittsburgh, the University of Tennessee, Memphis and the University of California, San Francisco.  Genomic DNA was extracted from buffy coat sampling using the PUREGENE® DNA Purification Kit at baseline.  Genotyping was undertaken at the Center for Inherited Disease Research (CIDR) using the Illumina Human1M-Duo BeadChip platform in 2009.  Sample exclusion criteria includes sample failure (call rate < 97%), genotypic-sex to reported-sex inconsistencies estimated based on X chromosome heterozygosity estimates, and the presence of first degree relative clusters based on autosomal identity by state analyses (probands from clusters were kept for study).  Genotyping was successful in a total of 2802 participants, of which 1663 were determined to be of European ancestry and 1139 were of African American Ancestry based on multi-dimensional scaling analyses in PLINK using HapMap release 22 populations as a reference.  SNPs with call rates of < 97%, HWE p-values < 1E-5 and minor allele frequencies < 1% were excluded prior to imputation.

Imputed genotypes were added to the dataset using MACH (v.1.0.16) with HapMap release 22 CEU haplotypes as a reference for the imputation.  Imputed genotypes were filtered based on MACH RSQR values of > 0.30 to exclude low quality SNPs.  Using high quality allele dosage data from MACH, linear regression models were implemented using MACH2QTL on the Biowulf Computational Cluster at the National Institutes of Health.  Linear regression models incorporated covariates of age at hematological assay (which occurred at year 3 of follow-up), study site, smoking status and additional covariates of C1 and C2 from multi-dimensional scaling analyses including only European ancestry samples.  Of the successfully genotyped European ancestry samples 237 participants were excluded for outlier status for one or more of the 6 hematological phenotypes and 351 were excluded due to missing data for at least 1 of the 6 phenotypes in this analysis leaving a final analytic sample size of 1075 participants with complete data.

*InCHIANTI*

The The Invecchiare in Chianti (InCHIANTI) Study is a population-based epidemiological cohort study in the Chianti region of Tuscany, Italy. The study employs two clinical sites, in the towns of Greve and Bagno a Ripoli (study site was used as an additional covariate in all analyses), with participants recruited from the population registries of these immediate areas. Further details on this cohort has been previously published elsewhere. Overnight fasted blood samples were used for genomic extraction and hematological analyses at baseline. Hematological assays were carried out using a Coulter LH 750 (Beckman Coulter, Instrumentation Laboratory, Milan, Italy), with fasting blood sample aliquots stored at -80C and thawed  at time of assay. DNA extracted from InChianti participants was genotyped at the Laboratory of Neurogenetics, National Institute on Aging, using Illumina 550K beadchips. After standard QC measures, genotypes were imputed using MACH (v.1.0.16) and linear regressiona nalyses were conducted using MACH2QTL.

*The Rotterdam Study I (RS) and The Rotterdam Study II (RSII)*

The Rotterdam Study is a prospective population-based cohort study that addresses determinants and occurrence of cardiovascular, neurological, ophthalmologic, psychiatric, and locomotor diseases in the elderly. Genetic components of the Rotterdam Study were divided into RS and RSII cohorts. RS was utilized in the discovery phase of analyses while RSII contributed data to the replication phase. These studies have been described in detail elsewhere. Venous blood samples were drawn from non-fasting participants. White blood cell and lymphocyte counts were assessed immediately after blood collection with an automated blood cell counter (Coulter Counter T660) using Beckman reagents.

Genotyping was conducted using the Illumina 550K array among self-reported Caucasian individuals. Samples were filtered for call rates at a minimum of 97.5% success. We excluded subjects for excess autosomal heterozygosity, mismatch between called and phenotypic gender, or being outliers identified by the IBS clustering analysis. SNPs were excluded for minor allele frequency < 1%, Hardy-Weinberg equilibrium P value<10-6, or SNP call rate < 90%. Genotypic analyses were conducted using ProbABEL.

*Heart and Vascular Health (HVH) Methods*

Setting and study design: The setting for this study was Group Health (GH), a large integrated health care system in western Washington State. Data were utilized from an ongoing case-control study of incident myocardial infarction (MI) and stroke cases with a shared common control group. Methods for the study have been described previously and are briefly summarized below.

The study was approved by the human subjects committee at GH, and written informed consent was provided by all study participants.

All study participants were GH members and aged 30-79 years. Cases were identified from computerized hospital discharge abstracts and billing records. Controls were a random sample of GH members frequency matched to MI cases on age (within decade), sex, treated hypertension, and calendar year of identification. The index date for controls was a computer-generated random date within the calendar year for which they had been selected.

Data collection: Eligibility and risk factor information were collected by trained medical record abstractors from a review of the GH medical record using only data available prior to the index date and through a telephone interview. Medication use was ascertained using computerized GH pharmacy records. A venous blood sample was collected from all consenting subjects, and DNA was extracted from white blood cells using standard procedures.

Genotyping: Genotyping was performed at the General Clinical Research Center's Phenotyping/Genotyping Laboratory at Cedars-Sinai using the Illumina 370CNV [BeadChip](http://wildebeest.pbworks.com/BeadChip) system. Genotypes were called using the Illumina [BeadStudio](http://wildebeest.pbworks.com/BeadStudio) software. Samples were excluded from analysis for sex mismatch or call rate < 95%. The following exclusions were applied to identify a final set of 301,321 autosomal SNPs: call rate < 97%; HWE p-value < 10-5 > 2 duplicate errors or Mendelian inconsistencies (for reference CEPH trios), heterozygote frequency = 0, SNP not found in HapMap, and inconsistencies across genotyping batches.  Imputation was performed using BIMBAM with reference to [HapMap](http://wildebeest.pbworks.com/HapMap) CEU using release 22, build 36 using one round of imputations and the default expectation-maximization warm-ups and runs.

For GWAS analysis, linear regression was used to investigate the association of each SNP with the WBC traits, using R. We used linear additive models with robust standard errors and estimated risk for each additional copy of the variant allele. Covariates included in the regression model were age at blood draw, sex and smoking status. SNPs were excluded from analysis for variance on the allele dosage ≤0.01.

*The Italian Network on Genetic Isolates (INGI) cohorts*

The Val borbera (VB) Cohort is an isolated population of 1800 individuals form North West Italy recruited as having at least one grandparent from the valley (as determined by population genealogy).  The Friuli Venezia Giulia (FVG) cohort is composed of 6 small villages from the north east of Italy: San Martino del Carso, Resia, Erto-Casso, Illegio, Clauzetto, Sauris. These villages have all been isolated as suggested by their history and the presence of few surnames. The Carlantino (CARL) cohort was recruited from a village situated in the Puglia region located in the southern east of Italy. It was founded by relatively few families in the XVI century and it has been isolated ever since. Genotyping was conducted using illumina 370KQuad array and imputed using MACH (v.1.0.16) with HapMap release 22 CEU.

Kooperative Gesundheitsforschung in der Region Augsburg *(KORAF3 and KORAF4)*

The study population for the KORA (http://epi.gsf.de/kora-gen/) is an independent population based sample from the general population living in the region of Augsburg, Southern Germany, examined in the years 1994/95 (KORAF3). The KORAF4 survey, a follow-up survey of the same region was conducted in 1999/2001. The standardized examinations applied in the surveys have been described in detail elsewhere. The KORAF3 and KORAF4 samples do not overlap.

DNA was extracted from fresh blood, and was stored at -80°C. Hematological assays were performed on fresh venous EDTA-anticoagulated blood using an automatic blood counter (Beckman Coulter STKS).

Genotyping of KORAF3 samples was performed using Affymetrix 500K Array Set. For fine scale analysis genotypes were imputed for all polymorphic HapMap phase 2 SNPs using a Hidden Markov Model as programmed in MACH. For association testing of imputed genotypes, we used the program MACH2QTL.

Genotyping of KORAF4 GWAS samples was performed using Affymetrix Human SNP Array 6.0. For fine scale analysis genotypes were imputed for all polymorphic HapMap phase 2 SNPs using IMPUTE. For association testing of imputed genotypes, we used the software SNPTEST.

*SORBS*

***Sample.***

All subjects are part of a sample from an extensively phenotyped self-contained population from Eastern Germany, the Sorbs. At present, about 1000 Sorbian individuals are enrolled in the study. Sampling comprised unrelated subjects as well as families. Extensive phenotyping included standardised questionnaires for past medical history and family history, collection of anthropometric data and a 75g-Glucose-tolerance-test. 785 subjects were available for the present study. The study was approved by the ethics committee of the University of Leipzig and all subjects gave written informed consent before taking part in the study.

***Blood count measurements.***

Venous EDTA blood samples were analyzed by use of the haematology automated analyser Sysmex XE-2100.

***Genotyping and imputation.***

Genotyping was performed with Affymetrix 500K and Affymetrix 6.0 GeneArrays. As a reference panel for imputation, we used Phase II CEU HapMap individuals; we imputed genotypes to nearly 2.5 million HapMap SNPs by using IMPUTE v1.0.0. Associations were assessed in the additive mode of inheritance by use of SNPTEST. Genomic control adjustment was performed to account for potential population stratification and cryptic relatedness.

*TwinsUK*

The TwinsUK cohort (KCL, www.twinsuk.ac.uk) is an adult twin British registry shown to be representative of singleton populations and the United Kingdom population 6. Ethics approval was obtained from the Guy’s and St. Thomas’ Hospital Ethics Committee. Written informed consent was obtained from every participant to the study.

Venous blood was anticoagulated with EDTA and FBCs were performed using either an ADVIA 2120 Haematology System (Siemens Healthcare Diagnostics, Deerfield, IL, US) or a XE2100 automated haematology analyser (Sysmex, Kobe, Japan) (on average within 24 hours from venesection (range 20 - 30 hrs). The two instruments displayed differences in measurements range, with means (SD) of 9.69 (0.96) and 11.17 (1.03) respectively. Hence association analyses were adjusted for instrument type.

All samples were typed with Infinium assay (Illumina, San Diego, USA). Imputation of genotypes was carried out using the software IMPUTE. The following QC filters were applied prior to imputation: per sample call rate > 95% (130 SNPs excluded), heterozygosity <33% and >37%, MAF ≥ 1% (9489 SNPs excluded), HWE p-value ≥ 10-6 (1555 SNPs excluded) and per SNP call rate ≥ 95% (777 SNPs excluded). Genotypes with imputation posterior probability on individual genotype calls ≤ 0.9 were discarded. The number of SNPs that passed quality control was 2,434,545.

*UK Blood Services Donor Panel 1 (UKBS1)*

The UKBS collection of Common Controls is an anonymised collection of DNA samples from 3,000 healthy blood donors. The collection has been established by the three British Blood Services of England, Scotland and Wales as part of the Wellcome Trust Case Control Consortium study and 1,500 of the samples (panel 1) served as shared controls in this study. Venous blood was taken from the dry pouch (attached to whole blood donation set) and placed in EDTA containing tube which was used to perform full blood counts (FBCs) on a Beckman-Coulter GenS automated blood count analyser (Villapointe, France).

1,500 samples from the (UKBS panel 1) were genotyped using the Affymetrix 500K Gene Chip as part of the Wellcome Trust Case Control consortium. The following QC criteria were applied to the data: HWE test P-value ≥ 1x10^-6^ (11,407 markers excluded), per-SNP missingness ≥ 0.05 (19,272 markers excluded), MAF ≥ 0.05 (116,025 SNPs excluded) leaving 361,352 SNPs in the analysis. The genomic inflation factor in the remaining dataset was 1.006. Imputation of SNPs was performed using HapMap2 phased data and the software IMPUTE.

**FUNDING**

This research was made possible by NIA/NIH contract AG000932-02 (2009) Characterization of Normal Genomic Variability.  This study utilized the high-performance computational capabilities of the Biowulf Linux cluster at the National Institutes of Health, Bethesda, MD [http://biowulf.nih.gov].

A portion of this research was facilitated by the recently-funded CHARGE infrastructure grant (CHARGE consortium: gene discovery for CVD and aging phenotypes. -- HL105756).

The Age, Gene/Environment Susceptibility Reykjavik Study is funded by NIH contract N01‐AG‐12100, the NIA Intramural Research Program, Hjartavernd (the Icelandic Heart Association) and the Althingi (the Icelandic Parliament).

The Atherosclerosis Risk in Communities Study is carried out as a collaborative study supported by National Heart, Lung, and Blood Institute contracts N01-HC-55015, N01-HC-55016, N01-HC-55018, N01-HC-55019, N01-HC-55020, N01-HC-55021, N01-HC-55022, and grants R01HL087641, R01HL59367 and R01HL086694; National Human Genome Research Institute contract U01HG004402; and National Institutes of Health contract HHSN268200625226C.  The authors thank the staff and participants of the ARIC study for their important contributions.  Infrastructure was partly supported by Grant Number UL1RR025005, a component of the National Institutes of Health and NIH Roadmap for Medical Research.

The National Heart, Lung, and Blood Institute's Framingham Heart Study is a joint project of theNational Institutes of Health and Boston University School of Medicine and was supported bythe National Heart, Lung, and Blood Institute's Framingham Heart Study (contract No. N01‐HC‐25195) and its contract with Affymetrix, Inc. for genotyping services (contract No. N02‐HL‐6-4278). Analyses reflect the efforts and resource development from the Framingham Heart Study investigators participating in the SNP Health Association Resource (SHARe) project. A portion of this research was conducted using the Linux Cluster for Genetic Analysis (LinGA‐II) funded by the Robert Dawson Evans Endowment of the Department of Medicine at Boston University School of Medicine and Boston Medical Center.

The Health ABC Study was supported in part by the Intramural Research Program of the NIH, National Institute on Aging, NIA contracts N01AG62101, N01AG62103, and N01AG62106. The genome-wide association study was funded by NIA grant 1R01AG032098-01A1 to Wake Forest University Health Sciences and genotyping services were provided by the Center for Inherited Disease Research (CIDR). CIDR is fully funded through a federal contract from the National Institutes of Health to The Johns Hopkins University, contract number HHSN268200782096C.

The InChianti Study was supported as a "targeted project" (ICS 110.1RS97.71) by the Italian Ministry of Health, by the U.S. National Institute on Aging (Contracts N01‐AG‐916413, N01‐AG‐821336, 263 MD 9164 13, and 263 MD 821336) and in part by the Intramural Research Program, National Institute on Aging, National Institutes of Health, USA. David Melzer’s participation was supported in part by NIH grant R01 AG24233-01.

The Heart and Vascular Health (HVH) Study was funded by grants HL085251 and HL087652 from the National Heart, Lung and Blood Institute.

The INGI cohorts and their investigators would like to acknowledge the regions of Friuli Venezia Giulia region and Fondo Trieste, and funding from Health Ministry project RF-FSR-2007-647201, Fondazione Compagnia di San Paolo. Fondazione Cassa di Risparmio di Alessandria.

The KORA Augsburg studies were financed by the Helmholtz Zentrum München, German Research Center for Environmental Health, Neuherberg, Germany and supported by grants from the German Federal Ministry of Education and Research (BMBF). Part of this work was financed by the German National Genome Research Network (NGFN). Our research was supported within the Munich Center of Health Sciences (MC Health) as part of LMUinnovativ.

The GWAS database of the Rotterdam Study was funded through the Netherlands Organization of Scientific Research NWO (nr. 175.010.2005.011, 911.03.012) and the Research Institute for Diseases in the Elderly (RIDE). This study was supported by the Netherlands Genomics Initiative (NGI)/NWO project number 050 060 810 (Netherlands Consortium for Healthy Ageing). We thank Dr Michael Moorhouse, Pascal Arp, Mila Jhamai, Marijn Verkerk and Sander Bervoets for their help in creating the genetic database. We thank the laboratory technicians Jeannette M Vergeer - Drop, Bernadette H M van Ast - Copier, Andy A L J van Oosterhout, Sue Ellen Mauricia, Andrea J M Vermeij - Verdoold, Els Halbmeijer - van der Plas, Debby M S Lont, and Hasna Kariouh for their help in phenotype assessment. The Rotterdam Study is supported by the Erasmus Medical Center and Erasmus University, Rotterdam; the Netherlands organization for scientific research (NWO), the Netherlands Organization for the Health Research and Development (ZonMw), the Research Institute for Diseases in the Elderly (RIDE), the Netherlands Heart Foundation, the Ministry of Education, Culture and Science, the Ministry of Health, Welfare and Sports, the European Commission (DG XII), and the Municipality of Rotterdam. Janine Felix and Abbas Dehghan are supported by the Netherlands Organization for Scientific Research (NWO, VICI no.918-76-619).

The Twins UK study was funded by the Wellcome Trust; European Community’s Seventh Framework Programme (FP7/2007-2013)/grant agreement HEALTH-F2-2008-201865-GEFOS and (FP7/2007-2013), ENGAGE project grant agreement HEALTH-F4-2007-201413 and the FP-5 GenomEUtwin Project (QLG2-CT-2002-01254). The study also receives support from the Dept of Health via the National Institute for Health Research (NIHR) comprehensive Biomedical Research Centre award to Guy's & St Thomas' NHS Foundation Trust in partnership with King's College London. TDS is an NIHR senior Investigator. The project also received support from a Biotechnology and Biological Sciences Research Council (BBSRC) project grant (G20234). Additional support includes the Wellcome Trust grant 076113/C/04/Z, the Juvenile Diabetes Research Foundation grant WT061858, and by the National Institute of Health Research of England. We thank the staff from the Genotyping Facilities at the Wellcome Trust Sanger Institute for sample preparation, Quality Control and Genotyping led by Leena Peltonen and Panos Deloukas; Le Centre National de Génotypage, France, led by Mark Lathrop, for genotyping; Duke University, North Carolina, USA, led by David Goldstein, for genotyping; and the Finnish Institute of Molecular Medicine, Finnish Genome Center, University of Helsinki, led by Aarno Palotie.

S.K. Ganesh is supported by National Heart, Lung and Blood Institute grant P30HL101290. N. Soranzo is supported by Wellcome Trust (Core Grant Number 091746/Z/10/Z).

The participation of A. P. Reiner is supported by National Heart, Lung and Blood Institute grant R01 HL-071862.

Holger Prokisch's participation was supported by the German Federal Ministry of Education and Research (BMBF) funded German Center for Diabetes Research (DZD e.V.) and Systems Biology of Metabotypes (SysMBo #0315494A).

Financial support was received from the German Research Council (KFO-152), IZKF (B27) and the German Diabetes Association. We would like to thank Knut Krohn (Microarray Core Facility of the Interdisciplinary Centre for Clinical Research, University of Leipzig) for the genotyping/analytical support and Joachim Thiery (Institute of Laboratory Medicine, Clinical Chemistry and Molecular Diagnostics, University of Leipzig) for clinical chemistry services. We thank Nigel W. Rayner (WTCHG, University of Oxford, UK) for the excellent bioinformatics support. Dr. Inga Prokopenko and Dr. Vasiliki Lagou were partial funded through the European Community's Seventh Framework Programme (FP7/2007-2013), ENGAGE project, grant agreement HEALTH-F4-2007-201413.

**Acknowledgements from COGENT collaborators:**

**COGENT and CARe**: The authors acknowledge the essential role of the Continental Origins and Genetic Epidemiology Network (COGENT) Consortium in development and support of this manuscript. COGENT members participating in support of this manuscript include the **WHI,** **ARIC**, **CARDIA, JHS, HANDLS, Health ABC**, and **GeneSTAR.**

**CARe:** The authors wish to acknowledge the support of the National Heart, Lung, and Blood Institute and the contributions of the research institutions, study investigators, field staff and study participants in creating this resource for biomedical research.  The following parent studies contributed study data, ancillary study data, and DNA samples through the Broad Institute (N01-HC-65226) to create this genotype/phenotype data base for wide dissemination to the biomedical research community:

**Atherosclerosis Risk in Communities (ARIC)**: University of North Carolina at Chapel Hill (N01-HC-55015), Baylor Medical College (N01-HC-55016), University of Mississippi Medical Center (N01-HC-55021), University of Minnesota (N01-HC-55019), Johns Hopkins University (N01-HC-55020), University of Texas, Houston (N01-HC-55017), University of North Carolina (N01-HC-55018). Other NIH support contributing to the GWAS in ARIC are: R01HL087641, R01HL59367, R01HL86694, U01HG004402, and HHSN268200625226C.

**Coronary Artery Risk in Young Adults (CARDIA)**: University of Alabama at Birmingham (N01-HC-48047), University of Minnesota (N01-HC-48048), Northwestern University (N01-HC-48049), Kaiser Foundation Research Institute (N01-HC-48050), University of Alabama at Birmingham (N01-HC-95095), Tufts-New England Medical Center (N01-HC-45204), Wake Forest University (N01-HC-45205), Harbor-UCLA Research and Education Institute (N01-HC-05187), University of California, Irvine (N01-HC-45134, N01-HC-95100).

**Jackson Heart Study (JHS)**: Jackson State University (N01-HC-95170), University of Mississippi (N01-HC-95171), Tougaloo College (N01-HC-95172).

**Healthy Aging in Neighborhoods of Diversity across the Life Span Study (HANDLS):** This research was supported by the Intramural Research Program of the NIH, National Institute on Aging and the National Center on Minority Health and Health Disparities (intramural project # Z01-AG000513 and human subjects protocol # 2009-149). Data analyses for the HANDLS study utilized the high-performance computational capabilities of the Biowulf Linux cluster at the National Institutes of Health, Bethesda, Md. (http://biowulf.nih.gov).

**GeneSTAR:**This research was supported by the National Heart, Lung, and Blood Institute (NHLBI) through the PROGENI (U01 HL72518) and STAMPEED (R01 HL087698-01) consortia. Additional support was provided by grants from the NIH/National Institute of Nursing Research (R01 NR08153), and the NIH/National Center for Research Resources (M01-RR000052) to the Johns Hopkins General Clinical Research Center.

**WHI:** The WHI program is funded by the National Heart, Lung, and Blood Institute, National Institutes of Health, U.S. Department of Health and Human Services through contracts N01WH22110, 24152, 32100-2, 32105-6, 32108-9, 32111-13, 32115, 32118-32119, 32122, 42107-26, 42129-32, and 44221. The authors thank the WHI investigators and staff for their dedication, and the study participants for making the program possible. A listing of WHI investigators can be found at http://www.whiscience.org/publications/WHI_investigators_shortlist.pdf.

**Acknowledgements for RIKEN collaborators:**

We would like to thank all the staff of the Laboratory for Statistical Analysis at RIKEN for their technical assistance. This study was supported by Ministry of Education, Culture, Sports, Science and Technology, Japan.

**SUPPLEMENTARY REFERENCES**

1. Harris TB, Launer LJ, Eiriksdottir G, Kjartansson O, Jonsson PV, et al. (2007) Age, Gene/Environment Susceptibility-Reykjavik Study: multidisciplinary applied phenomics. Am J Epidemiol 165: 1076-1087.

2. Aulchenko YS, Struchalin MV, van Duijn CM (2010) ProbABEL package for genome-wide association analysis of imputed data. BMC Bioinformatics 11: 134.

3. (1989) The Atherosclerosis Risk in Communities (ARIC) Study: design and objectives. The ARIC investigators. Am J Epidemiol 129: 687-702.

4. Purcell S, Neale B, Todd-Brown K, Thomas L, Ferreira MA, et al. (2007) PLINK: a tool set for whole-genome association and population-based linkage analyses. Am J Hum Genet 81: 559-575.

5. Price AL, Patterson NJ, Plenge RM, Weinblatt ME, Shadick NA, et al. (2006) Principal components analysis corrects for stratification in genome-wide association studies. Nat Genet 38: 904-909.

6. Splansky GL, Corey D, Yang Q, Atwood LD, Cupples LA, et al. (2007) The Third Generation Cohort of the National Heart, Lung, and Blood Institute's Framingham Heart Study: design, recruitment, and initial examination. Am J Epidemiol 165: 1328-1335.

7. Lin JP, O'Donnell CJ, Jin L, Fox C, Yang Q, et al. (2007) Evidence for linkage of red blood cell size and count: genome-wide scans in the Framingham Heart Study. Am J Hematol 82: 605-610.

8. Yang Q, Kathiresan S, Lin JP, Tofler GH, O'Donnell CJ (2007) Genome-wide association and linkage analyses of hemostatic factors and hematological phenotypes in the Framingham Heart Study. BMC Med Genet 8 Suppl 1: S12.

9. Ferrucci L, Bandinelli S, Benvenuti E, Di Iorio A, Macchi C, et al. (2000) Subsystems contributing to the decline in ability to walk: bridging the gap between epidemiology and geriatric practice in the InCHIANTI study. J Am Geriatr Soc 48: 1618-1625.

10. Hofman A, Breteler MM, van Duijn CM, Janssen HL, Krestin GP, et al. (2009) The Rotterdam Study: 2010 objectives and design update. Eur J Epidemiol 24: 553-572.

11. Psaty BM, Heckbert SR, Atkins D, Lemaitre R, Koepsell TD, et al. (1994) The risk of myocardial infarction associated with the combined use of estrogens and progestins in postmenopausal women. Arch Intern Med 154: 1333-1339.

12. Psaty BM, Heckbert SR, Koepsell TD, Siscovick DS, Raghunathan TE, et al. (1995) The risk of myocardial infarction associated with antihypertensive drug therapies. JAMA 274: 620-625.

13. Klungel OH, Heckbert SR, Longstreth WT, Jr., Furberg CD, Kaplan RC, et al. (2001) Antihypertensive drug therapies and the risk of ischemic stroke. Arch Intern Med 161: 37-43.

14. Doring A, Gieger C, Mehta D, Gohlke H, Prokisch H, et al. (2008) SLC2A9 influences uric acid concentrations with pronounced sex-specific effects. Nat Genet 40: 430-436.

15. Wichmann HE, Gieger C, Illig T (2005) KORA-gen--resource for population genetics, controls and a broad spectrum of disease phenotypes. Gesundheitswesen 67 Suppl 1: S26-30.

16. Tonjes A, Zeggini E, Kovacs P, Bottcher Y, Schleinitz D, et al. (2010) Association of FTO variants with BMI and fat mass in the self-contained population of Sorbs in Germany. Eur J Hum Genet 18: 104-110.

17. Tonjes A, Koriath M, Schleinitz D, Dietrich K, Bottcher Y, et al. (2009) Genetic variation in GPR133 is associated with height: genome wide association study in the self-contained population of Sorbs. Hum Mol Genet 18: 4662-4668.

18. (2007) Genome-wide association study of 14,000 cases of seven common diseases and 3,000 shared controls. Nature 447: 661-678.
